# On the Use of Probe Liquids for Surface Energy Measurements

**DOI:** 10.1021/acs.langmuir.3c00910

**Published:** 2023-11-15

**Authors:** Bernette M. Oosterlaken, Adriaan van den Bruinhorst, Gijsbertus de With

**Affiliations:** Laboratory of Physical Chemistry, Department of Chemical Engineering and Chemistry, Eindhoven University of Technology, PO Box 513, 5600 MB Eindhoven, The Netherlands

## Abstract

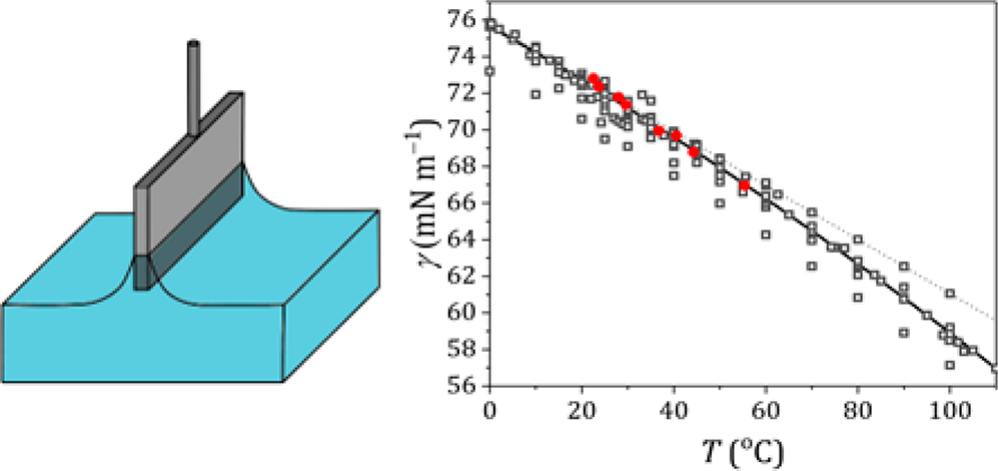

To assess the surface energy of solids, normally a set of probe liquids comprising polar and apolar compounds is used. Here we survey the surface tension of some frequently used probe liquids as given in the literature, for which a significant scatter appears to be present, and compare them with experimentally determined values. We discuss the influence of the liquid purity as well as the contact angle between the liquid and the Wilhelmy plate, which is commonly used for surface tension measurements. For hygroscopic polar probe liquids such as dimethyl sulfoxide, ethylene glycol, and formamide, water impurities appear to be of limited importance. Similarly, the amount of halogen impurities is of minor importance for diiodomethane and 1-bromonaphthalene, which decompose under the influence of light. Conversely, the influence of the contact angle for liquids that do not fully wet the plate, such as diiodomethane, is large in many cases, rendering a rather accurate determination of the contact angle necessary. Some discrepancies in the literature are indicated, and brief recommendations for future studies using such liquids are given.

## Introduction

The surface energy of a solid is notoriously difficult to assess. Generally, the surface energy of a solid is determined with a selection of probe liquids, for which surface tension γ_LV_ is well-known. From the measurements of the contact angle θ of a droplet on a solid the work of adhesion
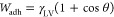
1for these liquids can be calculated. Although the work of adhesion can be established from these parameters, the inherent properties of the solid cannot be derived from this equation. The work of adhesion can also be written as
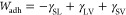
2in which γ_SL_ represents the interfacial energy between solid and liquid, γ_LV_ represents the surface tension of the probe liquid, and γ_SV_ represents the surface energy of the solid. Both γ_SL_ and γ_SV_ are unknown and cannot be determined from a single solid–liquid pair. Introducing another solid–liquid pair also leads to the introduction of a new unknown γ_SL_, such a set of equations can therefore never be solved uniquely.

Hence, another expression for the work of adhesion is needed. A suitable approach is the group contribution approach of which the Owens and Wendt^1^ and van Oss–Chaudhury–Good^2^ (vOCG) versions are often applied. The Owens–Wendt geometric mean method has been used frequently but often yields inaccurate results. This is due to the erroneous assumption that all polar materials interact with all other polar materials as a function of their internal polar cohesive forces.^3−5^ This leads, for example, to the wrong conclusion that ethanol is immiscible with water. In the vOCG model, the surface energy of a compound *i* is expressed by a dispersive, an acidic and a basic component given by
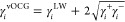
3The work of adhesion can now be described as
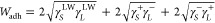
4γ_SL_ can be calculated by combining this equation with eq 1 if γ_LV_ and θ are measured and γ_L_^LW^, γ_L_^–^, and γ_L_^+^ values are taken from the literature. The values for γ_S_^LW^, γ_S_^–^, and γ_S_^+^ are supposed to be inherent properties of the solid and should not change when the solid is paired with different probe liquids. Thus, pairing the solid with at least three different probe liquids leads to a set of equations that can be solved uniquely.

The importance of having accurate contact angles to obtain surface energy values for solids was indicated by Białopiotrowicz.^6−9^ Recently, Burdzik et al.^10^ indicated that the uncertainty in the solid surface energy determined from the Owens–Wendt approach can be reduced considerably when using experimental surface tension values instead data from the literature. Similarly, an error analysis was done by Weng^11^ for the vOCG approach. This revealed the necessity of statistical consideration when evaluating and applying the multicomponent approach.

To be able to determine the properties of the solid as accurately as possible, it is therefore important that (i) the probe liquids used are chosen in such a way that all types of interactions (dispersive and acid–base) are properly represented, (ii) the experimentally acquired γ_LV_ values of the probe liquids match the literature values used for the vOCG parametrization,^2^ and (iii) contact angles are measured as accurately as possible. Moreover, as discussed later in this article, the surface condition is of the utmost importance for valid contact angle measurements. Proper characterization and/or equilibration is often not done, which might lead to significant errors if full wetting does not occur. Various reviews on contact angle measurements are available. They deal with aspects such as the fundamentals and application,^12^ performing reliable and reproducible advancing and receding contact angle measurements,^13^ assessing the quality of surfaces,^14^ and the effect of roughness.^15^

In this paper, we focus on the second point through the critical evaluation of the surface tension data for water, dimethyl sulfoxide, ethylene glycol, formamide, diiodomethane, 1-bromonaphthalene, and hexadecane, all probe liquids that are conventionally used for surface energy determination. We supplemented the literature data with new experimental data for probe liquids that were used as received. The surface tension of the selected probe liquids was determined via the widely applied Wilhelmy plate method. Using this method, a plate, generally made of roughened platinum–iridium, is hung on a balance above the liquid of interest. The plate is then immersed in the liquid and the surface tension determined via
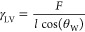
5in which *F* is the force measured by the balance, *l* is the wetted length, and θ_W_ represents the contact angle between liquid and Wilhelmy plate. Instead of assuming full wetting, we measured the θ_W_ for each of the probe liquids. The values obtained experimentally are compared with literature values. In a recent short opinion paper^16^ we already indicated the incoherency of many data. Details on the effects of impurities, purification, and condensation had to be omitted, but they are fully provided in this paper. Meanwhile, we discuss other aspects worth indicating when applying the well-established Wilhelmy method that are often overlooked and seldom reported, such as initial time effects, proper temperature measurement, condensation, and gas flow effects. Based on our observations, some considerations and suggestions for future research are given.

## Experimental Section

### Materials

Milli-Q water with a resistivity of 18 mΩ cm^–1^ was used. Formamide (GC, ≥99.5% purity), dimethyl sulfoxide (ACS reagent, ≥99.9% purity), ethylene glycol (anhydrous, 99.8% purity), and diiodomethane (ReagentPlus , 99% purity, copper stabilized) were acquired from Sigma-Aldrich (Merck KGaA, Darmstadt, Germany). *n*-Hexadecane (99% purity) was obtained from Acros Organics (Thermo Scientific Chemicals, Geel, Belgium). 1-Bromonaphthalene (95% purity) was acquired from TCI (Zwijndrecht, The Netherlands). Aluminum oxide (basic, activated, Brockmann I) was obtained from Honeywell Fluka (Fisher Scientific, Landsmeer, The Netherlands). Formamide, DMSO, and ethylene glycol were stored under an inert atmosphere until use. All reagents were used without purification, unless stated otherwise.

### Cleaning of Glassware

All glassware used for hexadecane, 1-bromonaphthalene, or diiodomethane was rinsed with water, ethanol, and hexane prior to use. Glassware used for formamide, DMSO, and ethylene glycol was first cleaned with water and then with ethanol. All glassware was dried with compressed air or left to dry in an oven at 100 °C before the measurements.

### Surface Tension Measurements

A Dataphysics DCAT 25 tensiometer equipped with a Wilhelmy plate was used to measure the surface tension. A Wilhelmy plate made of roughed platinum–iridium with dimensions of 10 × 19.9 × 0.2 mm^3^, resulting in a wetted length of 40.2 mm, was used. For each surface tension measurement, the probe liquid was added to a cylindrical glass container, which was placed inside the temperature-controlled Dataphysics TEC 250. The Pt100 temperature sensor was placed at the surface of the liquid. During the measurements of the hygroscopic liquids (formamide, DMSO, and ethylene glycol) an Ar flow was applied to minimize the water uptake, and the total timespan over which these three probe liquids were measured did not exceed 4 h. The Ar flow was measured with a Brooks R2-15-B flow meter (Porter Instrument Company). Before every new measurement, the platinum–iridium plate was burned hot to remove the solvent.

### Contact Angle Measurements

The wetting of the platinum–iridium plate was measured by using a Dataphysics OCA30 contact angle goniometer. The Wilhelmy plate was placed on top of a microscopy slide while ensuring that the plate was as flat as possible so that a proper baseline could be determined. The measurements of the hygroscopic liquids were performed inside a closed chamber under a nitrogen flow. For each measurement, six 2 μL droplets were placed on the platinum–iridium plate. Between measurements, the solvents were removed by burning the plate red hot.

### Condensation

To investigate the influence of condensation of a liquid on the platinum–iridium plate, two different tests were performed with water at 45 °C. In the first test, the plate was hung above the water surface. After the temperature had become constant, the weight as measured by the balance was noted every 2 min for a total duration of 45 min. This test was repeated twice. In the second test, the plate was immediately hung above the water surface, while the temperature was still increasing, and the weight was noted once every hour, for a little under 9 h. Before hanging the plate over the surface, the solvents were removed by burning it red hot.

### Purification of Probe Liquids

The purification of *n*-hexadecane was done with basic alumina, as reported by Goebel and Lunkenheimer.^17^ The glass column was cleaned with hexane and blown dry with compressed air. Then, the column was filled with 40 g of basic alumina on top of a layer of glass wool. After passing through the column, purified hexadecane was collected in a clean glass container. After the first 150 mL of purified hexadecane was collected, about 30 mL of purified hexadecane was collected for surface tension measurements. Subsequently, a container was placed to collect the rest of the purified hexadecane. This procedure was repeated three times in total.

## Results and Discussion

In this section, we report the data we have measured accompanied by some remarks about agreement with literature, while we deal with various other aspects in the discussion. We first determined the contact angle of all liquids on the platinum–iridium Wilhelmy plate (SI-1, Table S1). Except for diiodomethane, all liquids used showed complete wetting.

### Water

**Figure 1:**
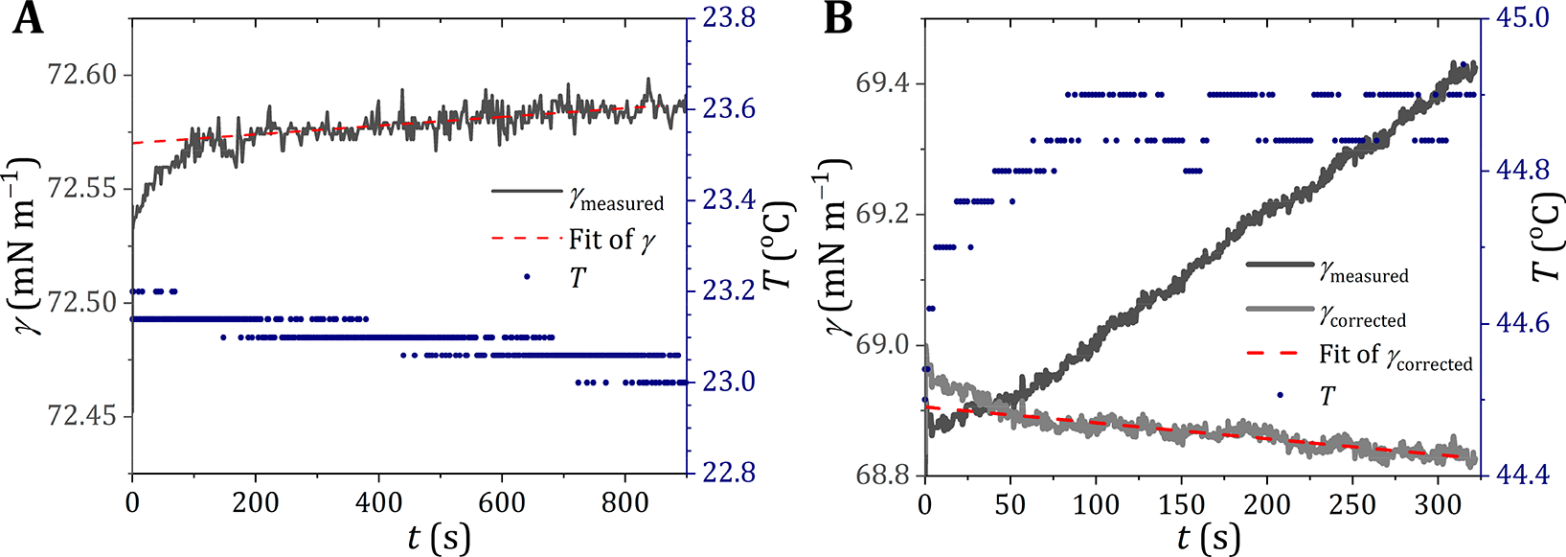
Surface tension measurements for Milli-Q water. Surface tension measurements (gray) and temperature (blue) as a function of time at a set temperature of (A) 25 °C and (B) 45 °C. The linear fit to determine the surface tension is given as a red dashed line. At higher temperatures, the observed surface tension increases in time as a result of condensation on the Wilhelmy plate, for which can be corrected (light gray line).

The surface tension of water was measured between ∼25 and ∼55 °C, and the results are given in Table S3 and Figure 1A,B, respectively.

When measuring at 25 °C, the surface tension first increases somewhat (0–200 s measuring time), after which a linear regime is reached with a slightly positive slope. By fitting a line through the linear regime and extrapolating to *t* = 0 s, the surface tension can be determined. When measuring at 45 °C, the surface tension also increases continuously, but with a larger slope. The latter effect is due to water condensation on the Wilhelmy plate, leading to an overestimation of the surface tension. By measurement of the weight of condensed material on a Wilhelmy plate that was hung above the water surface (SI-2, Figure S2), a correction can be made for the additional gravitational force on the Wilhelmy plate as a result of this condensation. Such correction leads to a surface tension evolution which becomes linear after approximately 60 s measurement time. Sometimes, at room temperature, also a small negative slope was observed. Whether a final small positive or negative slope occurs, probably depends on to the precise balance between evaporation and condensation. The surface tension obtained from the original surface tension measurement at *T* = 44.9 °C gives a value of 68.8 mN m^–1^ (data overlapping with corrected data), which is still in good agreement with literature values, although it is expected that for higher temperatures a larger deviation will occur as a result of condensation. The surface tension obtained from the original surface tension measurement and from the data corrected for condensation differs by approximately 0.1 mN m^–1^, which is similar to the sample standard deviation of the data. We therefore did not apply this correction for the measurements at other temperatures.

**Figure 2:**
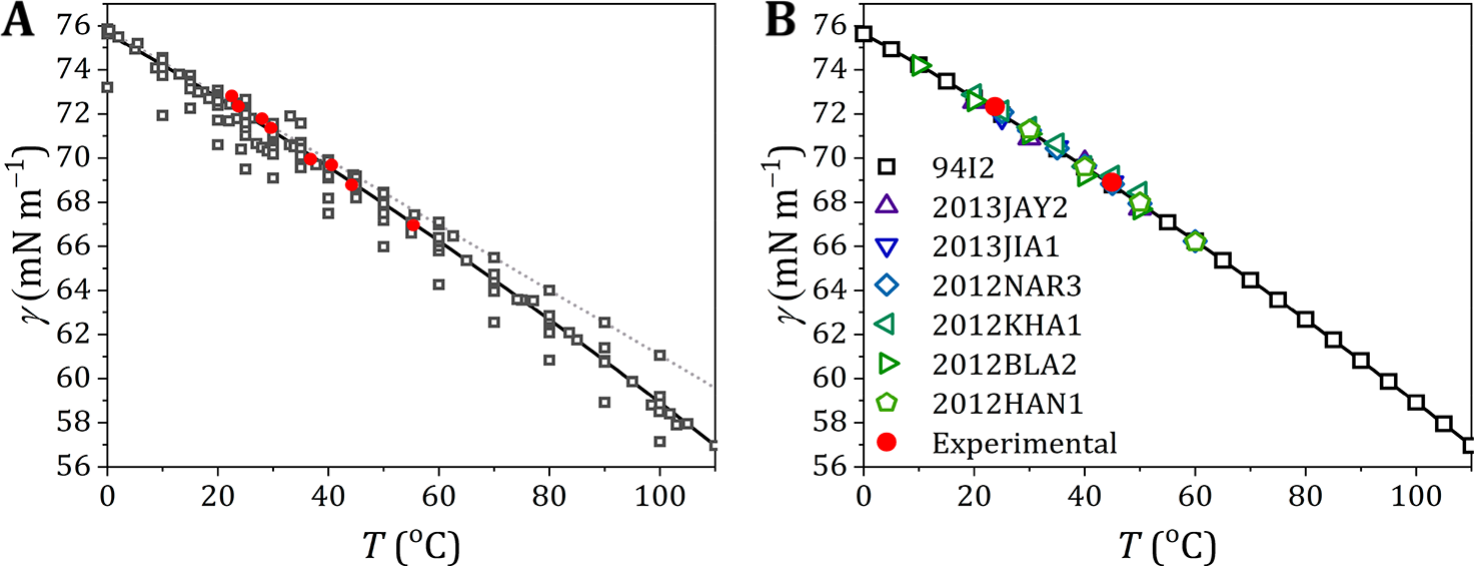
Surface tension of water. Surface tension of Milli-Q water as measured experimentally (red solid circles) compared to the literature values (open symbols) for different temperatures. (A) Complete overview of our experimental values compared to all literature values cited in this paper and the Supporting Information. Error bars for our experiments are smaller than the symbol used. (B) Selection of recent literature values compared with our experimental values. For clarity, only two experimental values are shown. More detailed overviews of the literature values are presented in Figure S4. The reference values and the corresponding abbreviations are taken from Landolt–Börnstein^24^ and the associated supplements.^25,26^ Note that reference 94I2^18^ is considered the reference for the surface tension of water according to the International Association for the Properties of Water and Steam (IAPWS), for which also a fitting line (black line) is included. The dotted gray line represents the fit from Jasper.^21^ References: 94I2,^18^ 2013JAY2,^27^ 2013JIA1,^28^ 2012NAR3,^29^ 2012KHA1,^30^ 2012BLA2,^31^ and 2012HAN1.^32^

The data obtained are in close agreement with the literature values (Figure 2 and Table S3). As a reference set for water, we used the International Association for the Properties of Water and Steam (IAPWS) data, described by
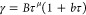
6where *B* = 235.8 mN m^–1^, μ = 1.256, and *b* = −0.625 and with
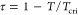
7with *T*_cri_ = 647.096 K (94I2,^18^ Vargaftik et al.^19^). Most published data agree reasonably well with the IAPWS reference data. One notable exception is the set obtained by Ramsay and Shields (1893R1^20^), using the capillary rise method, of which the data are systematically below the reference set.

Another exception is from the often-used Jasper data set,^21^ where water is represented by
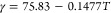
8with *T* in °C, yielding data that are systematically above the IAPWS data by a few tenths of a mN m^–1^ up to 40 °C, but rapidly increasing above 40 °C. The values from the expression used by Mulero et al.^22^ deviates less than 0.09 mN m^–1^ from the IAPWS data, but the expression contains 4 parameters instead of 3. Very recently, a proposal for an update of the IAPWS equations was presented.^23^ Although clear arguments were presented to do so, our overall conclusions for water are not affected.

### Polar Liquids

**Figure 3:**
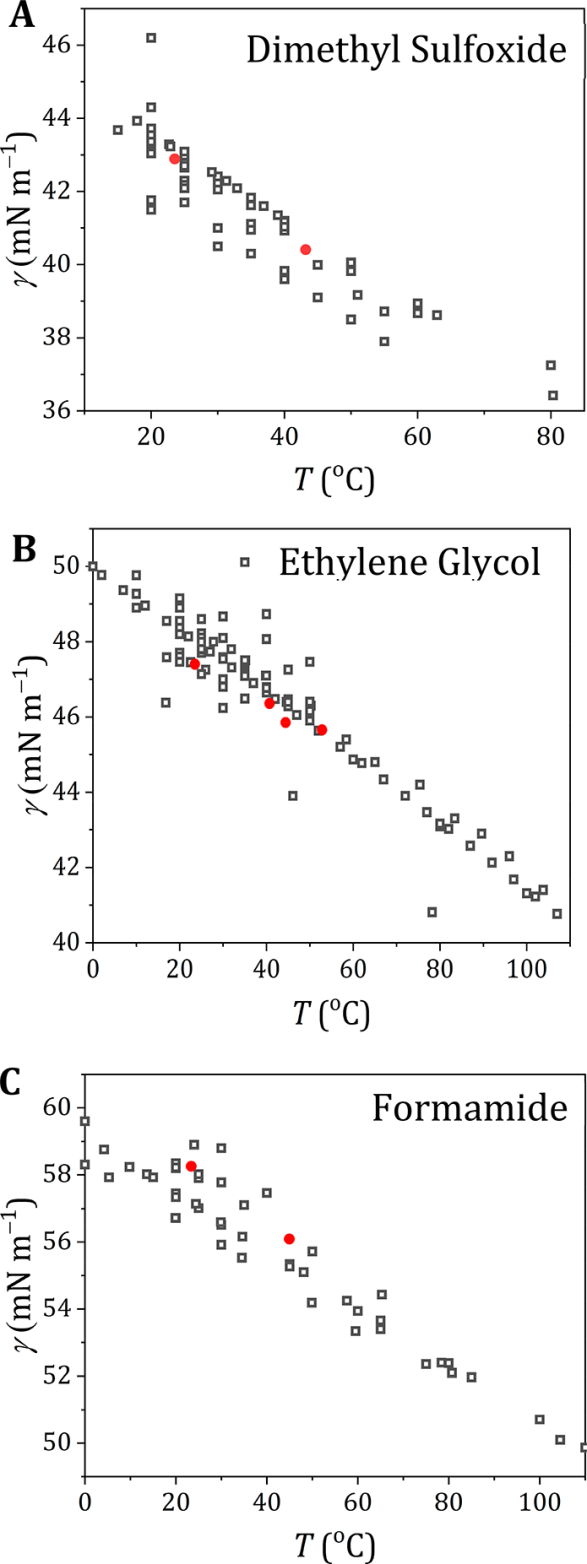
Surface tension of different polar probe liquids. Surface tension of dimethyl sulfoxide (A), ethylene glycol (B), and formamide (C) as measured experimentally (solid red circles) compared to the literature values as taken from Landolt–Börnstein^24^ and the associated supplements^25,26^ (open gray symbols) for different temperatures. Detailed overviews for the literature values are presented in Figure S9 (DMSO), Figure S10 (ethylene glycol), and Figure S11 (formamide).

The polar probe liquids dimethyl sulfoxide, ethylene glycol, and formamide were measured under Ar flow to prevent uptake of water during the surface tension measurements, as these are all hygroscopic (SI-5 and SI-7; Tables S4, S5, and S6 and Figures S6, S7, and S8, respectively). The experimentally observed surface tensions are compared to literature values in Figure 3.

For dimethyl sulfoxide the values agree well with the Jasper^21^ data (72J1) (maximum bubble pressure) and with the data sets 2013BAG1^33^ (Du Noüy ring) and 2013GEP1^34^ (pendant drop). Conversely, the data set 2000TSI1^35^ (Du Noüy ring) exhibits lower values than our data, and data set 1999KIN1^36^ (stalagmometer) follows a different temperature dependence.

For ethylene glycol the measured values are lower than those of data sets 72J1^21^ (capillary rise) and 87S1^37^ (capillary rise and Du Noüy ring), which both show a steeper temperature dependence compared to our experimental data. Data sets 2001JIM1^38^ (detaching drop) and 62S1^39,40^ (capillary rise and Du Noüy ring), on the other hand, are a close match to our data. It appeared that still other data are available, which we did not include.^41^. These data are in line with those presented here and do not change the overall picture.

For formamide our experimental data are higher compared to Jasper^21^ and are closer to data sets 2013SHU1^42^ and 31S2^43^ (both capillary rise). The temperature dependence of the surface tension of formamide seems steeper for our data compared to the literature values.

For both DMSO and ethylene glycol, our experimental values are in the middle of the cluster of the literature values, but for formamide, the observed surface tension seems to be a bit on the high side of the literature data.

### Apolar Liquids

**Figure 4:**
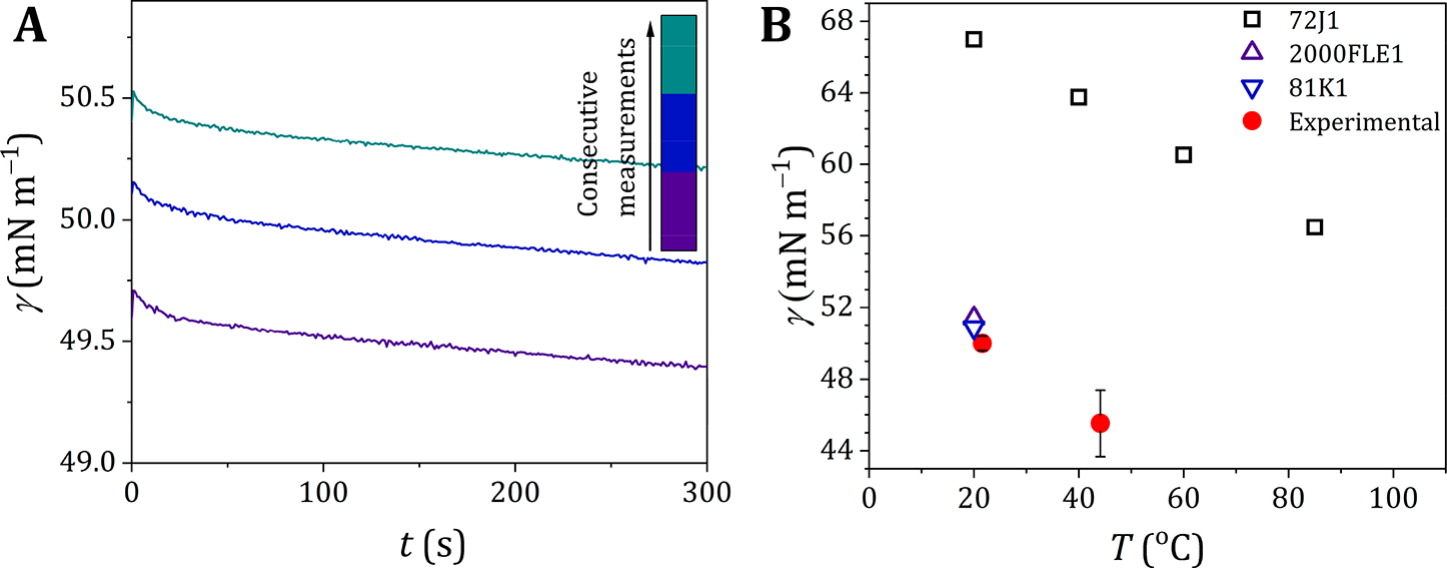
Surface tension of diiodomethane as a probe liquid. (A) Different surface tension measurements of diiodomethane as a function of time. (B) Surface tension of diiodomethane as measured experimentally (solid red circles; error bars represent the sample standard deviation) compared to the literature values (open symbols) and compared to the literature values as taken from Landolt–Börnstein^24^ and the associated supplements^25,26^ (open gray symbols) for different temperatures. References: 2000FLE1,^44^ 81K1,^48^ and 72J1.^21^

When the surface tension of the apolar probe liquids is measured, several issues occur. Diiodomethane, for example, is not fully wetting the platinum iridium plate. The contact angle of diiodomethane with the platinum iridium plate was determined to be 20.7° (SI-1, Table S1), and this contact angle was used in the Wilhelmy equation (eq 5) to determine the surface tension of diiodomethane (Figure 4).

The surface tension of diiodomethane seems to slightly increase with each consecutive measurement, resulting in a relatively high standard deviation (Figures 4A and S12A). On average, our data for the surface tension measured for diiodomethane are significantly different from the literature values (Figure 4B) and seem to have a much larger temperature dependence than data set 72J1.^21^ Both the data set 2000FLE1^44^ (Wilhelmy plate) and the data set from Jasper^21^ are listed in Landolt–Börnstein, but the latter one provides a much higher value. For a plausible explanation, see SI-10.^16^ Several other sources for diiodomethane could be found, but often it appeared that they referred to the same original sources, namely Fox and Zisman,^45^ Good and Elbing,^46^ or Fowkes,^47^ and we ignored these.

**Figure 5:**
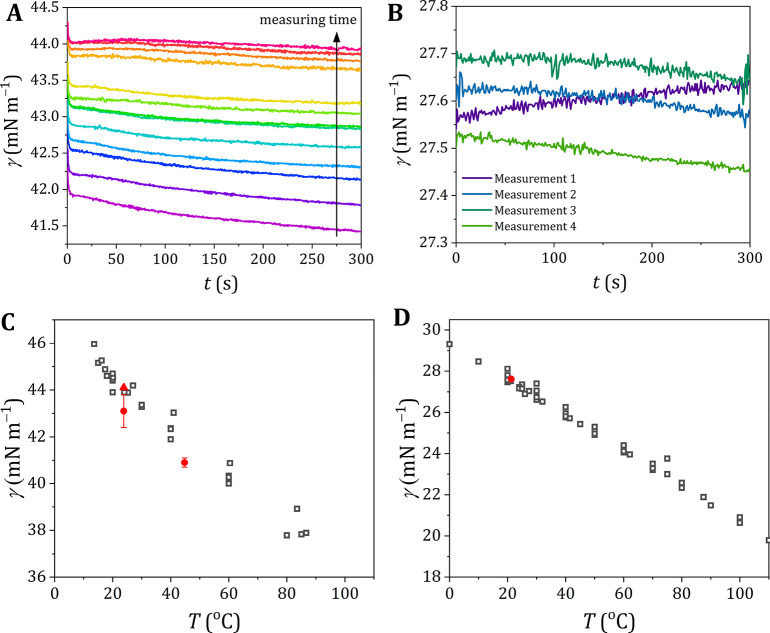
Consecutive surface tension measurements of 1-bromonaphthalene at 23.8 °C (A) and *n*-hexadecane at 21.2 °C (B). Surface tension of bromonaphthalene (C) and *n*-hexadecane (D) as measured experimentally (solid red circles; error bars indicate sample standard deviation for 13 measurements; the triangle in (C) indicates the equilibrium value obtained) compared to the literature values as taken from Landolt–Börnstein^24^ and the associated supplements^25,26^ (open gray symbols) for different temperatures. Detailed overviews of the literature values are presented in Figure S13 (1-bromonaphthalene) and Figure S14 (*n*-hexadecane).

Even though full wetting was observed for 1-bromonaphthalene, the surface tension increases somewhat for consecutive measurements (Figure 5A,B), but the curves converge over time (Figure 5A and Figure S15). For instance, at *T* = 23.8 °C the system seems to reach an equilibrium at γ_LV_ = 44.1 mN m^–1^, which is in closer agreement with the literature as compared to the average value (Figure 5C). At *T* = 44.7 °C, γ_LV_ = 40.9 mN m^–1^, which is more in agreement with the literature, although the value is still lower than the values reported in the literature. A tentative explanation for the small increase with time for the halogenated compounds is the evaporation of the halogens, thereby depleting the surface and increasing the surface tension.

For *n*-hexadecane, the measured surface tension is in good agreement with the literature values (Figure 5D). Summarizing this part, for all apolar liquids, the observed surface tension values are in the range of the literature values, but this range often shows a significant scatter.

In the following parts, we first discuss some generic issues encountered when applying the Wilhelmy plate method to measure surface tension and thereafter specific issues for each of the liquids used.

### Equilibrium and Temperature

Surface tension measurements suppose that an equilibrium is obtained. In our case, an approximate relaxation time of 200–400 s is observed. One possible explanation is that all liquids have a certain viscosity η, and the ratio between γ, η, and the relevant characteristic length λ determines the relaxation time for mechanical equilibrium is reached. Approximately, relaxation is described by an exponential with as relaxation time
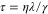
9Some authors take as characteristic length the capillary length, which is given by
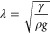
10in which ρ is the mass density and *g* the acceleration of gravity. For water, λ(H_2_O) = 2.7 mm. Other authors take the perimeter of the Wilhelmy plate,^49^ which equals 40.2 mm for our experiments. For the liquids considered and using the perimeter as the largest characteristic length, this results in relaxation times on the order of a millisecond. Hence, this effect is not responsible for the 200–400 s relaxation time observed in our experiments.

Rahman et al.^50^ studied the viscous relaxation effect experimentally, which resulted for liquids with a viscosity up to 1 Pa s in a relaxation time of maximally 1 min. Although the discrepancy with the theoretical estimate of Bracke et al.^49^ is large, it is still insufficient to explain the long relaxation times observed.

However, our temperature measurements indicate that the temperature equilibration is reached only after about 300 s. Furthermore, equilibration in the presence of an Ar flow leads to a significant decrease from the temperature set by the thermostat (SI-3, Figure S3B). For example, at a flow of about 4.6 SLPM (standard liters per minute), the difference with the set temperature is about −2.5 °C. Such a temperature decrease affects the observed surface tension easily by about 0.5 mN m^–1^, depending on the liquid. For example, for water, this would mean a difference in the surface tension of about 0.4 mN m^–1^. In view of the larger mass of the plate as compared with the thermocouple, this time will be longer for the whole setup, probably explaining the relaxation times observed. Proper temperature equilibration is, thus, a necessity. It seems that many experiments reported in the literature are done in a configuration where the temperature, as indicated by a thermostat, is used. This is not necessarily the correct temperature at the position of the surface tension measurement. Temperature measurement close to the measurement probe, as is used here, is thus a must. We also observed a larger scatter in the surface tension data of polar liquids for Ar flows above about 0.6 SLPM (SI-3, Figure S3A) due to the sensitivity of the balance to which the Wilhelmy plate is attached. In the present case the change in γ due to the temperature equilibration during the first 100–200 s is small, but this effect might be larger for other measuring configurations.

### Possible Issues When Measuring with the Wilhelmy Plate

The surface tension measurements described in this paper were performed via the Wilhelmy plate method. Although this method is in principle straightforward and reliable, some issues can occur, leading to deviations in the observed surface tension compared to the literature values. These factors are explained in more detail in the next sections.

### Condensation

As described in Condensation, condensation of the probe liquid on the Wilhelmy plate leads to an increased weight measured by the balance and thus an overestimation of the surface tension. Condensation indeed occurs for water, as demonstrated in Figure 1B, and this condensation effect is more pronounced at higher temperatures. As shown in the Experimental Section, the overestimation of the surface tension can be largely overcome by extrapolating the equation to *t* = 0. For water, an explicit correction for the increased weight due to condensation yielded surface tension values that are slightly (∼0.1 mN m^–1^) closer to the IAWPS reference data. However, it requires sufficient calibration experiments within the same temperature range as the surface tension measurements. The amount of condensation will depend on the precise measuring configuration and probably is less for larger measuring chambers.

### Gas Flow Effects

When hygroscopic polar probe liquids were measured in air, they could take up water during the measurement, possibly leading to an overestimation of the surface tension. In our case, to prevent water uptake during the measurement, the hygroscopic liquids were measured under an Ar flow. Upon increasing the Ar flow, the scatter of the data increases (SI-3). This is probably due to small movements in the Wilhelmy plate caused by the Ar flow. Smaller Ar flows do not lead to an increased scattering of the data. As indicated before, an Ar flow leads to a significant decrease in temperature, leading to a higher surface tension (SI-3). This again points to the importance of proper temperature equilibration and measurement.

### Partial Wetting and/or Pinning

When the liquid is not fully wetting the plate, the contact angle must be taken into account. Moreover, pinning could occur, which leads to further inaccuracy of the contact angle and thus of the surface tension. To prevent nonwetting (and possibly pinning) issues, the use of special chromatography paper (e.g., Whatman 1 CHR) instead of a platinum–iridium plate has been suggested previously.^51^ However, unfortunately, some experiments with water show that this approach is not as straightforward as it seems (SI-9). Not only does the chromatography paper suffer from curling upon exposure to the liquid, swelling of the paper when soaked in liquid makes determination of the paper dimensions prone to large error. Because of these issues, the test measurements with water did not result in reliable surface tension measurements (SI-9, Figure S16), and thus, this method was disregarded as a suitable alternative for the Pt–Ir Wilhelmy plate.

Various other methods to determine the surface tension of liquids are in use. We just mention the pendant drop method (Ramé–Hart advanced goniometer), drop count (stalagmometric) method, capillary rise method, and a more recent method due to Tadmor et al.^52^ The pendant drop method is highly dependent on the accuracy with which the shape of the droplet can be determined. For the capillary rise method, the shape of the meniscus must be determined so that a correction can be applied, but usually this correction factor is relatively small for thin capillaries. In the drop count method, one measures the weight of a drop of the fluid of interest that falls from a capillary. The Tadmor method employs a combination of centrifugation and gravity to manipulate normal and lateral forces such that the lateral components cancel each other, while the normal components are gradually increased. The Wilhelmy plate is in a certain sense the easiest method to apply in terms of executing the experimental procedure. Usually, full wetting of the plate is assumed, but one can in principle simply correct for this effect by incorporating the measured contact angle in the force equilibrium for the Wilhelmy plate equation (eq 5). The drawback is that in the regime of θ ≅ 20° to θ ≅ 40°, the contact angle must be rather accurately determined to prevent excessive errors on γ.

### Consistency of Water Data

In a recent opinion paper^16^ we indicated that the data for water as reported by Jasper (72J1^21^) and Ramsay and Shields (1893R2^20^) deviate from the IAPWS reference data. As a possible cause of the discrepancy, condensation was already mentioned. Condensation, however, is not the origin of the large differences between data sets 72J1^21^ (Figure S4I), 94I2^18^ (Figure 2A), and 1893R2^20^ (Figure S4J), as 72J1 and 1893R2 both use the capillary rise method to determine the surface tension. Although the data set of Ramsay and Shields was obtained more than a century ago, these authors provided a rather precise description of all of their experimental details. Despite that and the fact that the water was distilled twice, the reason for the discrepancy remains obscure (for a brief discussion, see ref (16)). Also, for the discrepancy of the water data, as given by Jasper with the reference data, the reason remains obscure. This is unfortunate because his reference set is often used in the physical–chemical literature.

The more recent data sets 2013JIA1,^28^ 2012NAR3,^29^ and 2012BLA2^31^ use the Wilhelmy plate method, but do not mention any condensation effects. Other methods presented in Figure 2B include the pendant drop method (data sets 2013JAY2^27^ and 2012HAN1^32^) and the drop count method (data set 2012KHA1^30^). Regardless of the method used, most of these more recent data sets are in close agreement with the standardized data as presented in data set 94I2.^18^

As is well-known, the surface tension of water is rather sensitive to impurities. To quote Hiemenz,^53^ “It is often noted that touching the surface of 100 cm^2^ of water with a fingertip deposits enough contamination on the water to introduce a 10% error in the value of γ. Not only must all pieces of equipment be clean, but also the experiments must be performed within enclosures or in very clean environments to prevent outside contamination”. See also Ponce-Torres and Vega.^54^ Therefore, such a water surface tension measurement can serve as a gauge to assess the quality of surface tension measurements whenever such data are reported.

### Water Contamination for Hygroscopic Liquids

As ethylene glycol, formamide, and dimethyl sulfoxide are hygroscopic, they will absorb water which might influence their surface tension value. Mixtures of these liquids with water (and 12 other liquids) were studied by Connors and Wright.^55^ These authors modeled the surface tension of mixtures on the basis of the basic assumptions that the organic component (component 2) exists in the surface phase in two states, free and bound (adsorbed), and that the number of binding sites for component 2 at the surface is proportional to the number of water molecules (component 1) in the surface. The resulting expression for the surface tension data is
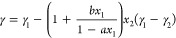
11where *x*_*j*_ is the mole fraction of component *j* and *a* and *b* parameters. Data for the liquids used are given in Table 1.

**Table 1:** Parameters *a* and *b* for the Connors–Wright Expression at 25 °C (Eq 11)^55^

	*a*	*b*
dimethyl sulfoxide	0.869	0.603
formamide	0.698	0.780
ethylene glycol	0.793	0.825

In our case, after measuring the surface tension, the water contents for dimethyl sulfoxide, ethylene glycol, and formamide were respectively 0.10, 0.10, and 0.17 mol % water (SI-4, Table S2). According to the Connors–Wright equation, this results in a negligible increase of the surface tension for all three liquids. Considering that the surface tension of water is much higher than any of the values for these three liquids, the enrichment of their surfaces with water is also expected to be small. Clearly, extensive exposure to air could lead to a larger water uptake and, consequently, a larger effect.

For all hygroscopic probe liquids, the literature values show significant scatter. The above considerations show that the scatter in the observed surface tension data is unlikely to be due to differences in the water uptake. But like for water itself, it could also arise due to the different methods that were used to measure the surface tension. Another reason might be the varying (organic) impurity contents. Only Körösi et al. (81K1)^48^ provide many details on the liquid used. Clever and Snead (63C1)^56^ indicate that for dimethyl sulfoxide a value higher by 0.3 mN m^–1^ was observed for a sample that as they stated “might contain as much water as 1.5 mol %”. According to the estimate indicated above, the water content in their sample was about 2.6 mol %. Generally, however, the information given in the literature is inadequate to assess the possible origin of these differences.

### Other Impurities

For the apolar liquids, next to incomplete wetting and pinning, other factors might play a role. For example, impurities are present in the probe liquids. Both diiodomethane and 1-bromonaphthalene are known to be photoactive compounds, reacting with UV-light,^57^ and the resulting iodine or bromine might affect the value obtained. Liquid I_2_ has γ = 36.9 mN m^–1^ at 125 °C,^58,59^ linearly extrapolating from the various data results in γ = 46.9 mN m^–1^ at 20 °C. As for diiodomethane the generally accepted value γ_LV_ = 50.8 mN m^–1^, some segregation of I_2_ might occur. If the extrapolated value for I_2_ applies, we can make an estimate for the mixture using the surface segregation considerations due to Kaptay et al.^60,61^ In a rather general way, the surface tension of solutions is described in terms of the partial surface tensions γ_A_ and γ_B_ of components A and B, both depending on the mole fractions *x*_A_ (*x*_B_) and surface concentrations *x*_A,s_ (*x*_B,s_) of both components and their surface areas ω_A_ and ω_B_, respectively. Choosing as independent variable *x*_B_ and a regular solution implementation, they are given by
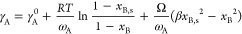

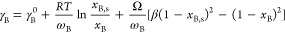
12where *RT* has its usual meaning, β represents the relative coordination number (we used β = 0.75), and Ω refers to the interchange energy (SI-11). The overall surface tension γ is defined in terms of the partial surface areas ω_A_ and ω_B_, partial surface tensions γ_A_ and γ_B_, and mole fractions *x*_A_ and *x*_B_ and given by
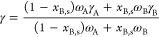
13Accepting this solution model, these equations must be solved iteratively when taking into account the difference in molar volume and an estimate of the interchange energy based on enthalpies of vaporization for both liquids and Berthelot’s rule. The required data for this model are listed in Table S11. The calculations showed that the decrease of γ_LV_ of diiodomethane for a small mole fraction *x* of I_2_ is given by Δγ_LV_ (mN m^–1^) = 50.8 – 2.1*x* (Figure S17). Such a small decrease cannot explain the discrepancy between the value of 61.8 mN m^–1^ as given by Jasper as one needs large amounts of I_2_ to reach 50.8 mN m^–1 ^^59^, or 50.88 mN m^–1^ as given by Körösi et al.^48^, or 51.4 mN m^–1^ as given by Fletcher and Nichols,^44^ assuming the same slope applies for a decrease from 61.8 mN m^–1^. Such a large amount is unlikely as our estimate clearly shows the minor influence of the I_2_ impurity, consistent with the remark of Fletcher and Nichols that the liquid–air surface tensions of fresh and aged samples were found to be identical. A plausible explanation for the discrepancy is given in ref (16).

We observed that diiodomethane is not fully wetting the plate. The contact angle θ appeared to be ∼41° in the case the plate was left for some time exposed to air and ∼32° in the case the plate was measured immediately after cooling from glowing red hot by a Bunsen burner. Furthermore, after prewetting the contact angle is ∼21°, which is actually the value to be used if the tensiometer dips the plate in the liquid before measuring. It should be noted that quite some deviations between the different contact angle measurements were present (see SI-1, Table S1) due to the somewhat unpredictable behavior of diiodomethane while depositing it for the contact angle measurement with the syringe and possibly also to pinning as sometimes the values measured on the left and right sides of the drop were rather different. Unfortunately, in this range the cosine of θ _W_ as used in eq 5 is rather sensitive to such deviations. A change of 1° for a θ_W_ of about 30°–40° changes the value for the surface tension by 1–2% (0.5–1.0 mN m^–1^). Our data are close to the values reported by Körösi et al.^48^ and Fletcher and Nichols.^44^

For 1-bromonaphthalene (∼44.4 mN m^–1^) and bromine^62^ (∼41.8 mN m^–1^) the surface tension values are closer. Using the same model as for diiodomethane leads to Δγ_LV_ = 44.4–1.0*x* for a small mole fraction *x* of Br_2_. Hence, the influence of any Br_2_ impurity is estimated to be even smaller than that for I_2_. As in our case, the surface tension of 1-bromonaphthalene gradually increased with time from about 41.9 to about 44.0 mN m^–1^; Br_2_ contamination is an unlikely cause. In view of the large vapor pressure of 0.3 atm for Br_2_ at room temperature, the data rather suggest that Br_2_ slowly evaporates from the solution, thereby raising the surface tension.

*n*-Hexadecane is not photoactive, but especially longer alkanes are often contaminated with surface active components,^17^ such as long-chain carboxylic acid impurities which may be the result of slow oxidation or of contamination.^63−67^*n*-Hexadecane was purified over basic alumina, as described by Goebel and Lunkenheimer,^17^ but this did not have the desired effect to decrease the variability within one measurement of the surface tension measurements. The reason for this is unclear.

In Table 2 the surface tension data as measured as well as those from the literature are summarized, illustrating once more the variety in values obtained for the same compound.

**Table 2:** Summary of Surface Tension Data^a^

compound	γ (mN m^–1^)	lit.	ref
water	72.4	72.4	Figure S4
dimethyl sulfoxide	42.9	43.3–41.9	Figure S9
ethylene glycol	47.4	47.1–49.0	Figure S10
formamide	58.2	56.8–59.0	Figure S11
diiodomethane	50.0	50.0–67.0	Figure 4
1-bromonaphthalene	43.1	43.8–44.2	Figure S13
*n*-hexadecane	27.6	27.5–28.0	Figure S14

a Full references given in the figure indicated. All data close to *T* = 23 °C.

## Conclusions

Using the well-established Wilhelmy plate, we re-evaluated the measurement of the surface tension method of seven probe liquids commonly used for the determination of the solid surface energy, i.e., water, formamide, dimethyl sulfoxide, ethylene glycol, diiodomethane, *n*-hexadecane, and 1-bromonaphthalene. The surface tension of water is relatively easy to assess with a high accuracy. Although somewhat different values can be found in the literature, most recent measurements are in close agreement with the standardized data set as presented by the IAPWS. For the other probe liquids, significant deviations between different measurements occur.

We highlight three aspects of the Wilhelmy plate measurements that are often omitted but can (partially) explain the observed deviations.

First, partial wetting of the Wilhelmy plate was observed for diiodomethane, with a contact angle varying with time. In principle, correcting the surface tension for partial wetting is straightforward. However, the contact angle of diiodomethane with plate surfaces exposed for some time to air becomes about 40°, exactly in the range where the largest accuracy is needed. For example, a difference of 1° in this range affects the surface tension by 2 mN m^–1^. A nonzero contact angle with a Wilhelmy plate thus influences the results tremendously.

Second, the plate and surrounding liquid may have a significantly lower temperature than indicated by the thermostat. This is particularly relevant when an Ar flow is used to prevent water uptake, even at moderate flows.

Finally, condensation on the Wilhelmy plate can be significant, especially when measured at higher temperatures. The effect of condensation can be corrected by subtracting the gravitational contribution of condensation from the measured force on the balance. At the temperatures (20–55 °C) explored in this work, such correction is small; the observed water surface tension values match those in the literature.

Other differences within the probe liquid data sets can be explained by the variety of measuring methods. Some of the artifacts as encountered for the Wilhelmy plate method could also have an effect in other methods. For example, impurities in a liquid always affect the outcome of a surface tension measurement regardless of the method used. As most solid surface energies will be calculated with surface tension data obtained from “as received” or “as stored” probe liquids, we explored the influence of typical contaminants on the measured surface tension. For the polar and hygroscopic probe liquids (ethylene glycol, dimethyl sulfoxide, and formamide), water uptake during the measurement hardly affected the results. Diiodomethane and 1-bromonaphthalene decompose under the influence of light and produce I_2_ and Br_2_, respectively. It was shown that these decomposition impurities also hardly influenced the surface tension measurements. For hexadecane a purification was suggested in the literature,^17^ but this did not lead to less variation. Although purity is to be pursued, purification steps may also introduce unexpected contaminations that affect the final measurement to a larger extent than the initial impurities.

To return briefly to the solid surface energy, it might be fruitful to consider other liquids to determine solid surface properties instead of the conventionally employed ones. Diiodomethane is considered a “standard” in the surface tension and contact angle measurements, especially in combination with the vOCG theory. However, the reference values at 20 °C differ more than 15 mN m^–1^.^16^ Possible apolar alternatives include tetrachloromethane, octane, dodecane, and 1,4-dioxane. Tetrachloromethane is formally considered apolar, although the C–Cl bonds individually have strong dipole moments. An advantage is that its properties are well-known. Octane is a solvent commonly used in inverse gas chromatography, and BET surface area measurements performed at room temperature. It is probably a better choice than hexane, another common solvent for IGC, because it suffers more from persistent isomer impurities^69^ that are introduced during the distillation of raffinate oil (composed of C6 alkane mixtures). Dodecane has been used and appears to be a good choice. 1,4-Dioxane has a smaller polarity (dipole moment μ = 0.45 D) compared to water (μ = 1.85 D) and does have much less ability to form hydrogen bonds. Admittedly, we did not use these liquids as this became only clear in a rather late stage of this work. Looking in the literature, many surface tension data are reported, but most of these liquids have not been used to assess surface energy values. We therefore would like to encourage the exploration of other liquids than the conventionally used ones for the determination of the solid surface energy.

We note Weng^11^ has discussed the errors involved in the determination of the solid surface energy by the vOCG method when the most widely used liquid triplets are employed. However, nowadays it is often advocated that more than three liquids should be used, though it is not straightforward to select a proper set of probe liquids. Moreover, it is also clear that relatively small errors can lead to large errors in the solid surface energy.

In conclusion, although surface tension measurements appear to be straightforward, they are loaded with issues. Some of these issues can be taken into account properly, such as partial wetting, humidity, and other impurity effects. Unfortunately, generally, the information on the liquids used as given in the literature is inadequate to assess the possible origins of differences in results.

We recommend for future work that independently of what liquid is used, a measured value for the surface tension of water at room temperature is given so that readers can assess the quality of the other measurements reported. This should include the average value, the standard deviation (either of the sample or in the mean), and the number of measurements such that other researchers can use these data in their statistical comparisons. Information given about purity is usually rather limited but should be as complete as feasible. To avoid rapid contamination by volatile organic compounds, the top liquid layer can be discarded with a clean Teflon pipet just before the contact angle measurements. In this way, a clean and pure surface of the liquid can be obtained. For other methods, see ref (68). Moreover, a temperature measurement close to the measuring probe (e.g., plate and ring) is required, as this temperature may differ considerably from the thermostat set point, and the temperature equilibration might take a few minutes. Also, reporting the relative humidity for all experiments is desirable as well as stating whether full or partial wetting occurred. If partial wetting is noticed, the contact angle must be determined as accurately as possible and reported in a similar way to the surface tension. With the above considerations and an extended set of probe liquids, we think that the Wilhelmy plate method can be successfully employed to quantify the solid surface energy of materials.

## Supporting Information Available

Contact angles, correcting for condensation on the Wilhelmy plate, effect of argon flow on the surface tension measurements, drying hygroscopic liquids, surface tensions measured, accuracy of data, literature data for the surface tension of water, surface tension data for the hygroscopic liquids, chromatography paper experiments, an explanation for the diiodomethane data discrepancy, data for the surface tension calculation of solutions (PDF)
