# Supplementary material for: On the Use of Probe Liquids for Surface Energy Measurements
Source: Langmuir. 2023 Nov 15;39(47):16701–11. doi: 10.1021/acs.langmuir.3c00910 (PMC10688178; doi:10.1021/acs.langmuir.3c00910)
Supplement: Supplementary file 1 — la3c00910_si_001.pdf [file la3c00910_si_001.pdf]

Supplementary Information

for

On the Use of Probe Liquids for Surface Energy Measurements

Bernette M. Oosterlaken, Adriaan van den Bruinhorst and Gijsbertus de With*

Laboratory of Physical Chemistry, Department of Chemical Engineering and Chemistry, Eindhoven University of Technology, PO Box 513, 5600 MB, Eindhoven, the Netherlands.

*G.deWith@tue.nl

SI-1: Contact angles

For all liquids the contact angle on the Wilhelmy plate was determined. Figure S1 shows a schematic of the setup and an image of the actual setup. Except for diiodomethane, full wetting of the plate was observed. Consequently, a value of 0° was used for the contact angle θ_W. The data for diiodomethane are given in Table S1.

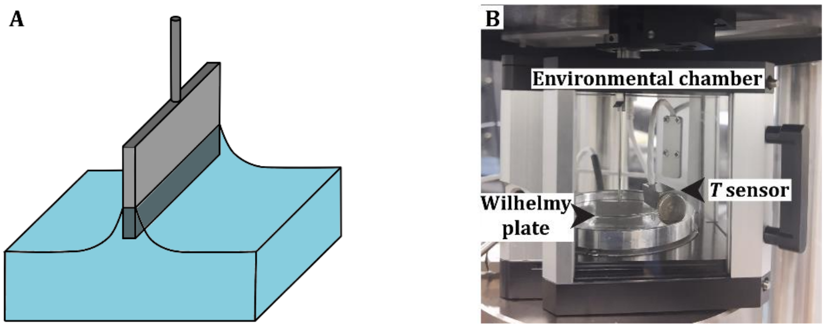

Figure S1: A) Schematic of the Wilhelmy plate setup; B) Image of the actual setup showing the Wilhelmy plate, the temperature sensor and the environmental chamber.

Table S1: Contact angles of diiodomethane on the platinum–iridium Wilhelmy plate.

Probe liquid		Contact angle θ (°)	*n*
Diiodomethane	After exposure of the plate to air	40.83 ± 0.85 [a)]	6
	90 s after glowing red hot[b)]	32.22 ± 3.98	24
	Discarding data with $\Delta\theta > 2°$ [c)]	32.02 ± 0.44	18
	After wetting the plate with diiodomethane	20.28 ± 4.07	40
	Discarding data with $\Delta\theta > 2°$ [c)]	20.69 ± 3.64	30

[a)] ± denotes the sample standard deviation. [b)] Time required to cool down. [c)] This value is arbitrary but seems reasonable in view of the experimental scatter.

The data show more than average scatter as usual for contact angle measurements. Nevertheless, for all data measured directly after glowing and after wetting the plate with diiodomethane prior to measuring and discarding the measurements for which the difference in

contact angle was more than 2°, the average is virtually the same as the complete set (Table S1). As the surface tension measurements are performed by first immersing the plate in the probe liquid, the contact angle of the prewetted plate should be used. In the main text we therefore used $\theta = 20.7°$.

SI-2: Correcting for condensation on the Wilhelmy plate

To determine the contribution of condensation on the overestimation of the surface tension, the weight of condensed material was measured over time by hanging the Wilhelmy plate just above the water surface. The weight was noted every two minutes during three consecutive measurements (Figure S2).

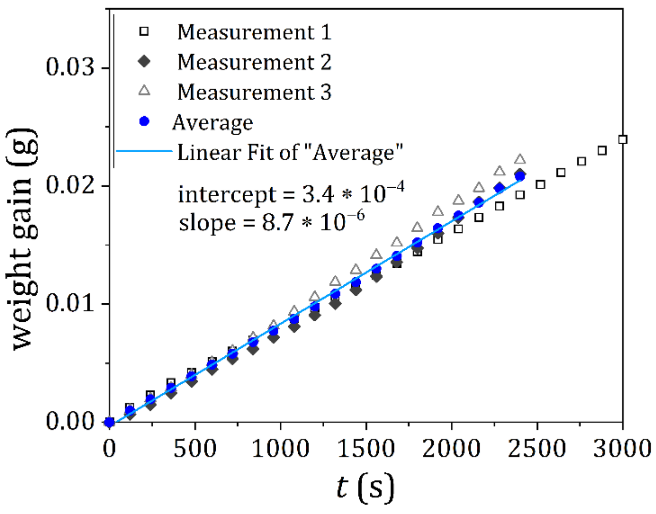

Figure S2: Condensation of water on the Wilhelmy plate at T = 45 °C.

The surface tension is normally determined via:

$$\gamma = \frac{F}{l \cdot \cos(\theta_W)} \quad \text{(S1)}$$

in which F represents the force on the balance of the tensiometer due to the surface tension, l represents the wetted length of the Wilhelmy plate and θ_W is the contact angle.

In the case of condensation on the plate, a higher weight is registered by the balance in the tensiometer. This additional gravitational force should be subtracted from the force that is exerted on the balance by the surface tension to obtain the corrected surface tension:

$$\gamma_{corr} = \frac{F - F_z}{l \cdot \cos(\theta_W)} \quad \text{(S2)}$$

in which F, l, and θ_W are defined as above, and F_z corresponds to the additional gravitational force. This gravitational force can be calculated for each time point via:

$$F_z = m \cdot g = (a \cdot t + b) \cdot g \quad \text{(S3)}$$

in which m represents the mass of the condensed material and g represents the gravitational constant. The weight gain caused by condensation of water on the platinum iridium plate was recorded every 2 minutes. Three measurements were performed (grey symbols), after which the

average weight gain was determined (blue circles). Using a linear fit on the average weight gain F_z (light blue line), the contribution of condensation on the Wilhelmy plate was determined, as calculated from the slope a and intercept b for the fit of the average weight gain (Figure S1). Both non-corrected and corrected surface tension data are shown in Figure 1B (main text).

SI-3: Effect of argon flow on the surface tension measurements

For the hygroscopic liquids, an argon flow was applied to prevent the uptake of water. To investigate the influence of the argon flow on the results, the surface tension of water was measured under large and small argon flows (Figure S3).

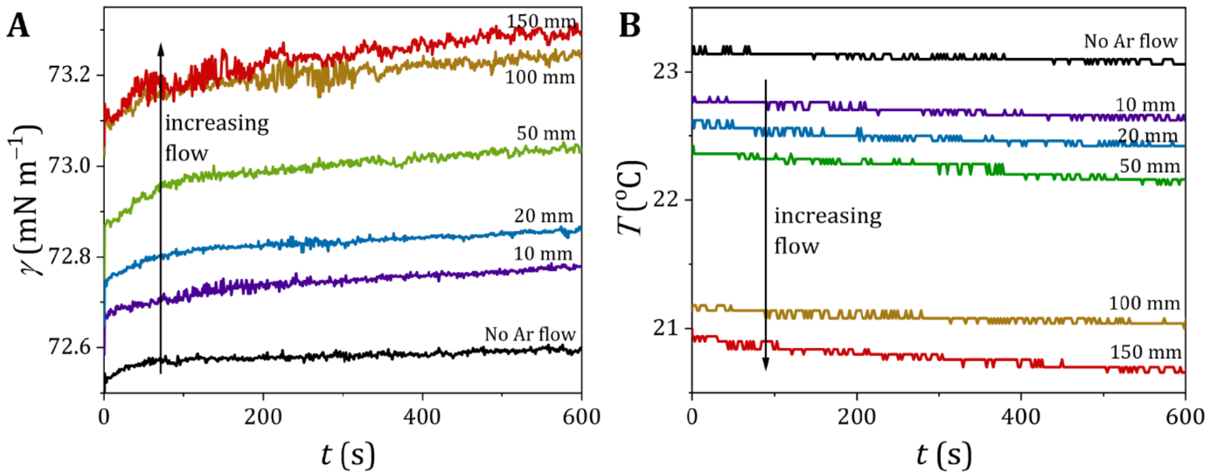

Figure S3: Fluctuations in surface tension resulting from an argon flow during measurement. Surface tension (**A**) and temperature (**B**) with different argon flows applied during the measurements. Different colors indicate different measurements. Colors used in the temperature plot correspond to those used in the surface tension plot.

As can be seen in Figure S3, a large argon flow leads to more scatter of the data, possibly because the argon flow is causing slight movements in the plate that affect the measurement. A smaller argon flow does not show such scatter. For the large flow a temperature decrease of about 2 °C was observed. However, even a small argon flow leads to a temperature decrease of the water of 1 about °C, resulting in a higher surface tension value. Hence, more systematic measurements using a more quantitatively measured flow using a Brooks R2-15-B (Porter Instrument Company) flow meter were done as well. A similar temperature decrease of more than 2 °C results for the maximum flow applied (150 mm sphere height corresponding to 4.6 standard liter per minute flow). Positioning the thermocouple as close as possible to the Wilhelmy measuring position without affecting the curvature near the Wilhelmy plate is thus advised.

SI-4: Drying hygroscopic liquids

For drying, mol sieves were used that were first activated by heating at 380 °C for 8 h. Ethylene glycol was dried using 3 Å mol sieves (CAS # 1318-02-1, MERCK) and dimethyl sulfoxide using 4 Å mol sieves (CAS # 1318-02-1, MERCK). Liquid was poured over the mol sieves until it just covered the mol sieves and were let drying for 2 weeks. Thereafter the mol sieve-liquid mixture was sieved to retrieve the dried liquid. The amount of water was determined with

Karl-Fischer titration[1-2] using a Metrohm 899 with a generator electrode without diaphragm under an argon atmosphere. CombiCoulomat fritless (MERCK) was used as titration liquid. The detection limit was 10 µg ± 0.5 %. Table S2 shows the water content as determined.

Table S2: Water content analysis.

	Before drying (ppm water)	After drying (ppm water)	After γ measurement (ppm water)	(mol%)
Dimethyl sulfoxide	9200 ± 290	23 ± 1	233 ± 8	0.10
Ethylene glycol	913 ± 22	23 ± 3	289 ± 9	0.10
Formamide	-	87 ± 9	692 ± 1	0.17

SI-5: Surface tensions measured

For all liquids examined, below the experimentally determined surface tension data are given. In the tables given below n indicates the total number of measurements comprising both repeated immersions and new samples.

Table S3: Surface tension data for water.

T (°C)	Sample st. dev. σ_T (°C)	γ (mN m^{-1})	Sample st. dev. σ_γ (mN m^{-1})	n
22.5	0.20	72.83	0.12	3
23.7	0.07	72.36	0.04	6
28.0	0.04	71.80	0.10	4
29.6	0.02	71.37	0.08	6
36.8	0.08	69.95	0.09	3
40.6	0.25	69.69	0.06	5
44.2	0.57	68.79	0.14	3
55.4	0.85	66.96	0.14	8

Table S4: Surface tension data for dimethyl sulfoxide.

T (°C)	Sample st. dev. σ_T (°C)	γ (mN m^{-1})	Sample st. dev. σ_γ (mN m^{-1})	n
23.5	0.06	42.85	0.12	5
43.5	0.54	40.41	0.10	6

Table S5: Surface tension data for ethylene glycol.

T (°C)	Sample st. dev. σ_T (°C)	γ (mN m^{-1})	Sample st. dev. σ_γ (mN m^{-1})	n
23.5	0.06	47.42	0.03	5
40.7	0.40	46.36	0.20	4
44.4	0.03	45.85	0.01	3
52.8	0.41	45.65	0.09	4

Table S6: Surface tension data for formamide.

T (°C)	Sample st. dev. σ_T (°C)	γ (mN m^{-1})	Sample st. dev. σ_γ (mN m^{-1})	n

23.3	0.11	58.18	0.06	5
45.1	0.26	56.09	0.17	7

Table S7: Surface tension data for diiodomethane.

T (°C)	Sample st. dev. σ_T (°C)	γ (mN m^{-1})	Sample st. dev. σ_γ (mN m^{-1})	n
21.6	0.04	50.00 [a)]	0.40	3
44.1	0.02	45.53 [a)]	1.86	5

[a)] when using 20.7 as the contact angle.

Table S8: Surface tension data for 1-bromonaphthalene.

T (°C)	Sample st. dev. σ_T (°C)	γ (mN m^{-1})	Sample st. dev. σ_γ (mN m^{-1})	n
23.8	0.09	43.11	0.72	13
44.7	0.03	40.75	0.17	4

Table S9: Surface tension data for n-hexadecane.

T (°C)	Sample st. dev. σ_T (°C)	γ (mN m^{-1})	Sample st. dev. σ_γ (mN m^{-1})	n
21.2	0.10	27.61	0.08	4

SI-6: Accuracy of data

Accepting that a Gauss distribution represents the outcome of measurements properly, all relevant characteristics can be calculated once the expectation value for the mean μ of the parent distribution or the average $\bar{x}$, the expectation value for the parent distribution standard deviation σ or the sample standard deviation s and the number of measurements n are given[3]. To be explicit, using $\mathscr{E}(\cdot)$ for expectation value, $\mathscr{V}(\cdot)$ for variance and $\mathscr{S}(\cdot) = [V(\cdot)]^{1/2}$ for standard deviation, we have

$$\mathscr{E}(x) = \mu \quad \text{and} \quad \mathscr{E}(s^2) = \sigma^2$$

where

$$\bar{x} = \mathscr{E}(x) = \sum\nolimits_{j=1}^{j=n} x_j \Big/ n \quad \text{and} \quad s^2 = \mathscr{V}(x) = \sum\nolimits_{j=1}^{j=n} (x_j - \bar{x})^2 \Big/ (n-1)$$

with x_j the j-th measurement. The variance $\mathscr{V}(\bar{x})$ of the average $\bar{x}$ and the variance $\mathscr{V}(s^2)$ of the variance s^2 are given by

$$\mathscr{V}(\bar{x}) = s^2/n \;\text{ and }\; \mathscr{V}(s^2) = \mathscr{E}\{[\mathscr{E}(s^2) - s^2]^2\} = n^{-1}\left(\mu_4 - \frac{n-3}{n-1}\mu_2^2\right)$$

where μ_n is the nth central moment of the parent distribution. For a Gauss distribution with $\mu = 0$, $\mu_4 = \mu_2^2 = 3\sigma^4$ and $\mu_2 = \sigma^2$. Although $m_2 = n^{-1}\Sigma_j(x_j - \bar{x})^2$ is an estimator for the moment μ_n, it is biased, while s^2 is a (nearly) unbiased estimate. They are related by $s^2 = nm_2/(n-1)$.

To estimate the standard deviation of s, we use that for a function $f(x)$ of x [4],

$$\mathcal{V}[f(x)] = \left[\frac{\mathrm{d}f(x)}{\mathrm{d}x}\right]^2_{x=\theta} \mathcal{V}(x)$$

so that, for $s = f(s^2) = (s^2)^{1/2}$,

$$\mathcal{V}(s) = \left[\frac{\mathrm{d}f(s^2)}{\mathrm{d}s^2}\right]^2_{s=\sigma} \mathcal{V}(s^2) = \left[½(\sigma^2)^{-1/2}\right]^2_{s=\sigma} \mathcal{V}(s^2)$$

For a Gauss distribution therefore $\mathcal{V}(s^2)$ becomes

$$\mathcal{V}(s^2) = 2\sigma^4/(n-1)$$

so that for the standard deviation $\mathcal{S}(s)$ of s we have

$$\mathcal{S}(s) = \sqrt{\frac{1}{4\sigma^2}\frac{2\sigma^4}{n-1}} = \left[\frac{1}{2(n-1)}\right]^{1/2} \sigma \cong \left[\frac{1}{2(n-1)}\right]^{1/2} s$$

where in the last step we replaced σ by s, because clearly the exact value of σ is unknown. A more precise and complex analysis, using that the variance has a X^2-distribution, leads to

$$\mathcal{S}(s)' = s\frac{\Gamma[(n-1)/2]}{\Gamma(n/2)} \cdot \sqrt{\left(\frac{n-1}{2} - \left[\frac{\Gamma(n/2)}{\Gamma[(n-1)/2]}\right]^2\right)}$$

Table S10 provides some numbers from which we conclude that $\mathcal{S}(s)$ is sufficiently accurate.

For a typical surface tension measurement, we have $\bar{x}$ = 40 mN m^{-1}, s = 0.5 mN m^{-1} and n = 5. Hence, $s_{\bar{x}} \cong 0.5/5^{1/2}$ mN m^{-1} = 0.22 mN m^{-1} or $s_{\bar{x}}/\bar{x}$ = 0.028. Similarly, $\mathcal{S}(s) \cong [1/2{\cdot}4)^{1/2}{\cdot}0.5$ = 0.18 mN m^{-1} or $S(s)/s$ = 0.36. Their relative values thus differ by a factor of about 10. Hence, it is probably fair to say to that while the average and its associated standard deviation are rather well determined by a relatively small number of measurements, the sample standard deviation and its associated standard deviation are not so well determined. It should also be kept in mind that because the distribution for s^2 is a X^2-distribution, which is a skewed distribution.

Table S10: Relative standard deviations $\mathcal{S}(s)$ and $\mathcal{S}(s)'$ of the sample standard deviation s of n data.

n	2	4	6	8	10	20	30
$\mathcal{S}(s)/s$	0.707	0.408	0.316	0.267	0.236	0.162	0.131
$\mathcal{S}(s)'/s$	0.756	0.422	0.323	0.272	0.239	0.163	0.132

SI-7: Literature data for the surface tension of water

In order to be able to assess the variability of surface tension data for the liquids examined, we provide plots of the surface tension as a function of temperature, including the data measured. The data are largely from Landolt-Börnstein[5] and the associated supplements[6-7]. Individual references are given ion the various captions.

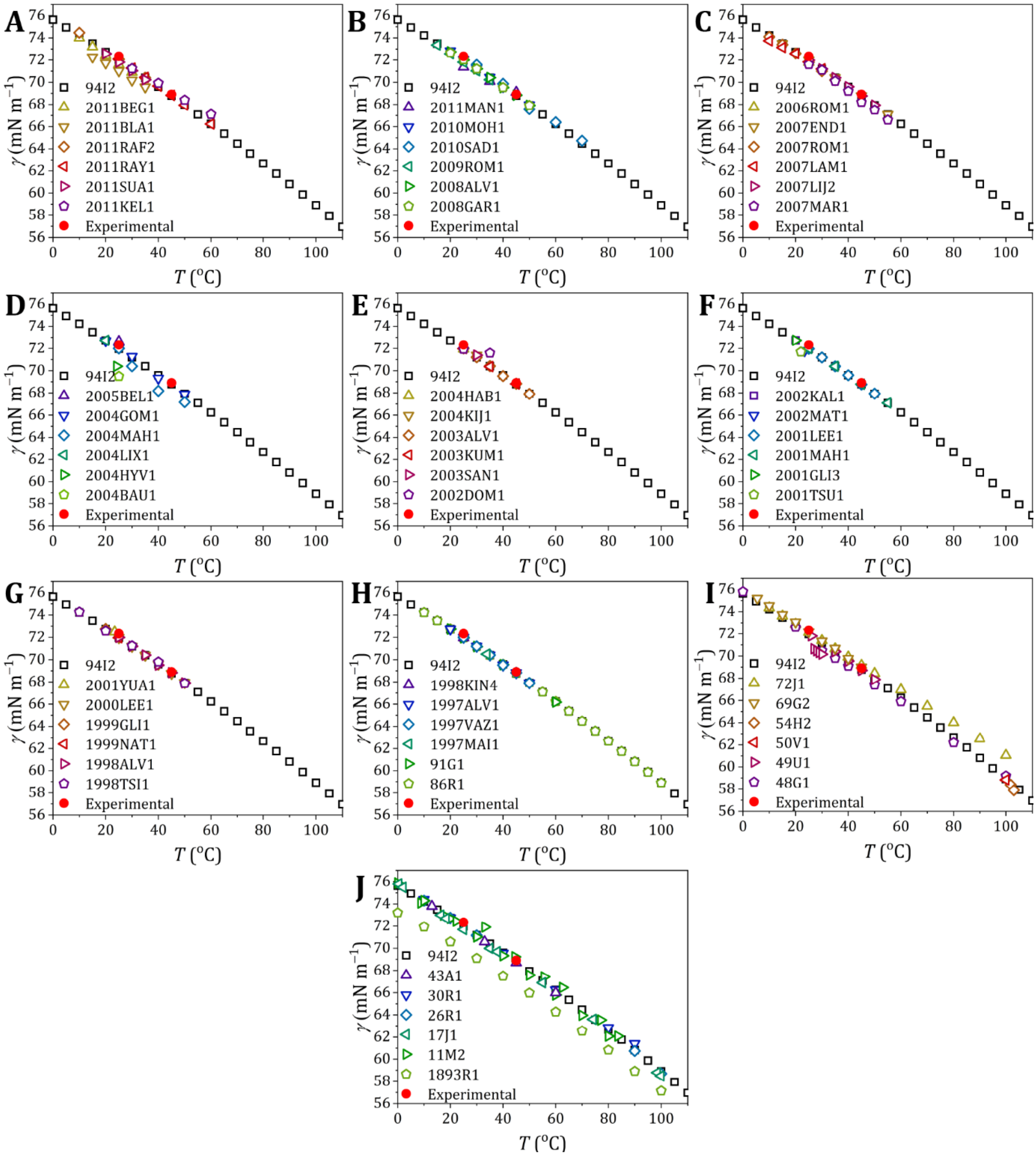

Figure S4: Surface tension of water. Surface tension of MilliQ water as measured experimentally (red solid circles) compared to literature values (open symbols) for different temperatures. The reference values and the corresponding abbreviations are taken from Wohlfahrt[5] and the later supplements[6-7]. Note that reference 94I2[8] is considered the reference standard according to the International Association for the Properties of Water and Steam (IAPWS). References: **A.** 2011BEG1[9], 2011BLA1[10], 2011RAF2[11], 2011RAY1[12], 2011SUA1[13], 2011KEL1[14]; **B.** 2011MAN1[15], 2010MOH1[16], 2010SAD1[17], 2009ROM1[18], 2008ALV1[19], 2008GAR1[20]; **C.** 2006ROM1[21], 2007END1[22], 2007ROM1[23], 2007LAM1[24], 2007LIJ2[25], 2007MAR1[26]; **D.** 2005BEL1[27],

2004GOM1[28], 2004MAH1[29], 2004LIX1[30], 2004HYV1[31], 2004BAU1[32]; **E.** 2004HAB1[33], 2004KIJ1[34], 2003ALV1[35], 2003KUM1[36], 2003SAN1[37], 2002DOM1[38]. **F.** 2002KAL1[39], 2002MAT1[40], 2001LEE1[41], 2001MAH1[42], 2001GLI1[43], 2001TSU1[44]. **G.** 2001YUA1[45], 2000LEE1[46], 1999GLI1[47], 1999NAT1[48], 1998ALV1[49], 1998TSI1[50]. **H.** 1998KIN1[51], 1997ALV1[52], 1997VAZ1[53], 1997MAI1[54], 91G1[?], 86R1[55]. **I.** 72J1[56], 69G2[57], 54H2[?], 50V1[58-59], 49U1, 48G1[60]. **J.** 43A1[?], 30R1[?], 26R1[61], 17J1[62], 11M2[63], 1893R1[64]. References 91G1, 54H2, 43A1 and 30R1 are irrecoverable from the information provided.

As mentioned in the main text, the data from Ramsay and Shields are systematically lower as compared to the IAPWS data. Shifting their values up by 2 mN m^{-1} reduces the average deviation with the IAPWS data to about 0.15 mN m^{-1}. For other liquids their data do not deviate as much as for water, but generally also have a negative deviation: Figure S5 shows data on diethyl ether, methyl formate, ethyl acetate, carbon tetrachloride and ethanol. From the comparison as given in Figure S5, we suggest that the value given by Ramsay and Shields for methyl formate at 70 °C contains a typographic error. From their (detailed) experimental description no clear reason could be distilled what is the possible reason for these discrepancies. Noteworthy is that most researchers measure the surface tension of a liquid against air while Ramsay and Shields measured against the saturated vapor. This effect is, however, small for the pressure involved, that is, about 1 bar. Nevertheless, the striking resemblance after shifting and the, so it seems, generally negative deviation suggest some systematic error.

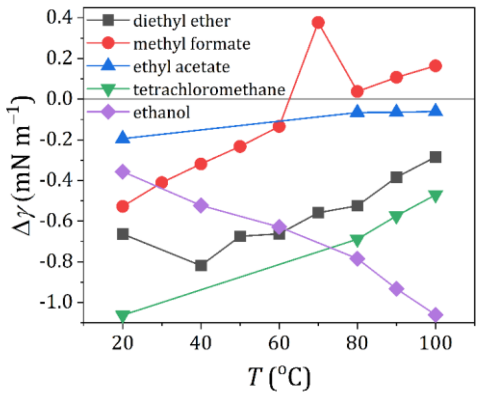

Figure S5: The deviation of the Ramsay and Shields (RS)[65] data from present literature data: Diethyl ether (Bi[66]), methyl formate (Mulero[67]), ethyl acetate (Mulero[67]), tetrachloromethane (Jasper[56]) and ethanol (Jasper[56]).

SI-7: Surface tension data for the hygroscopic liquids

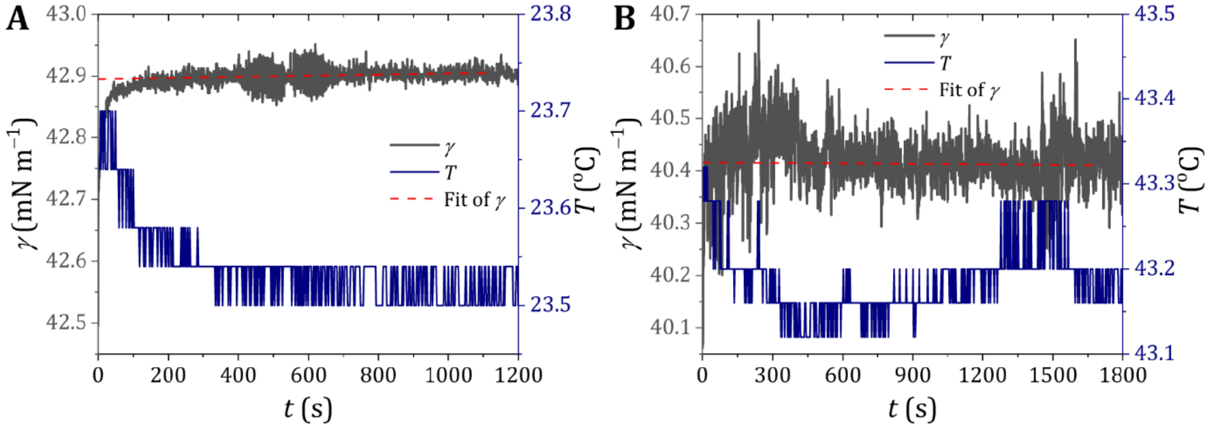

Figure S6: Surface tension of dimethyl sulfoxide (DMSO). Examples of surface tension measurements (grey) and temperature (blue) as a function of time at (**A**) 25 °C and (**B**) 45 °C. The linear fit to determine the surface tension is given as a red dashed line. The measurements were performed under argon flow to prevent uptake of water.

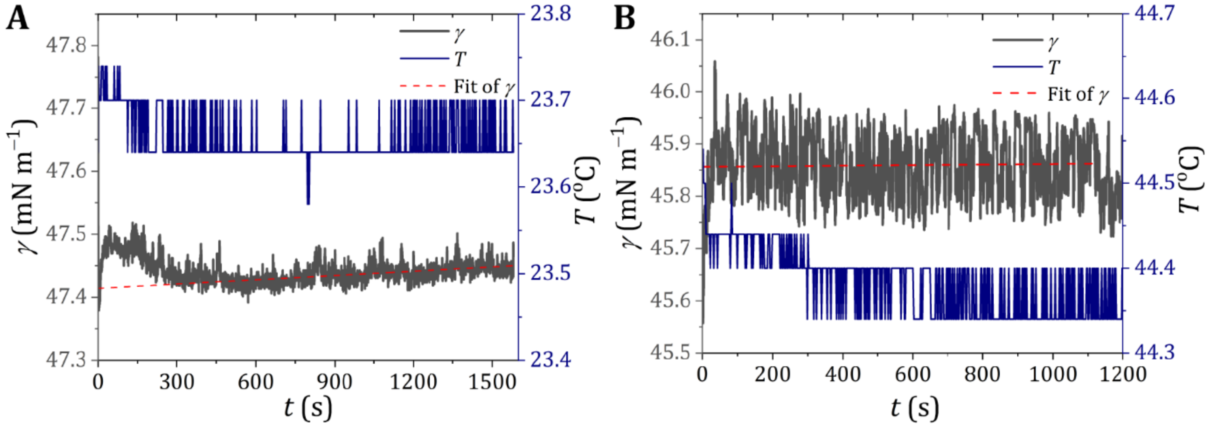

Figure S7: Surface tension of ethylene glycol. Examples of surface tension measurements (grey) and temperature (blue) as a function of time at (**A**) 25 °C and (**B**) 45 °C. The linear fit to determine the surface tension is given as a red dashed line. The measurements were performed under argon flow to prevent uptake of water.

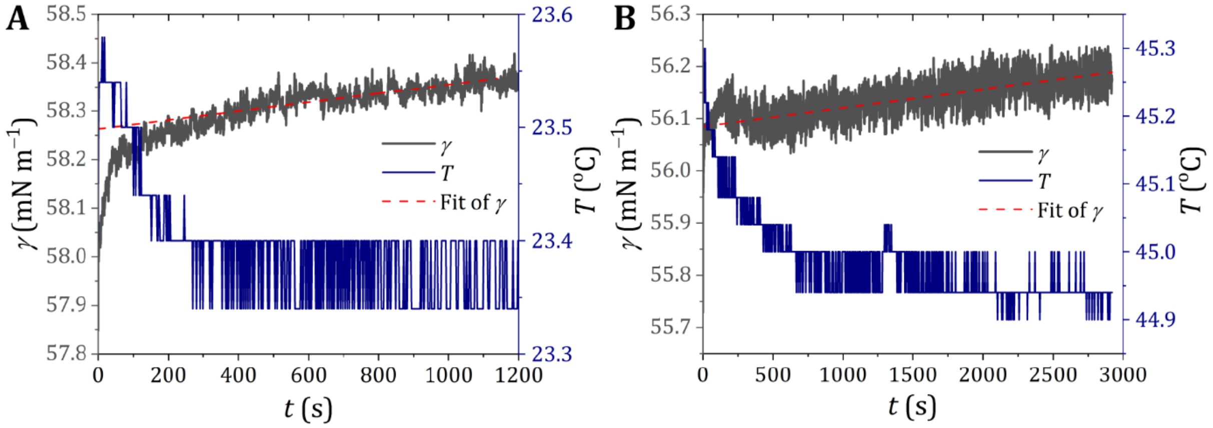

Figure S8: Surface tension of formamide. Examples of surface tension measurements (grey) and temperature (blue) as a function of time at (**A**) 25 °C and (**B**) 45 °C. The linear fit to determine the surface tension is given as a red dashed line. The measurements were performed under argon flow to prevent uptake of water.

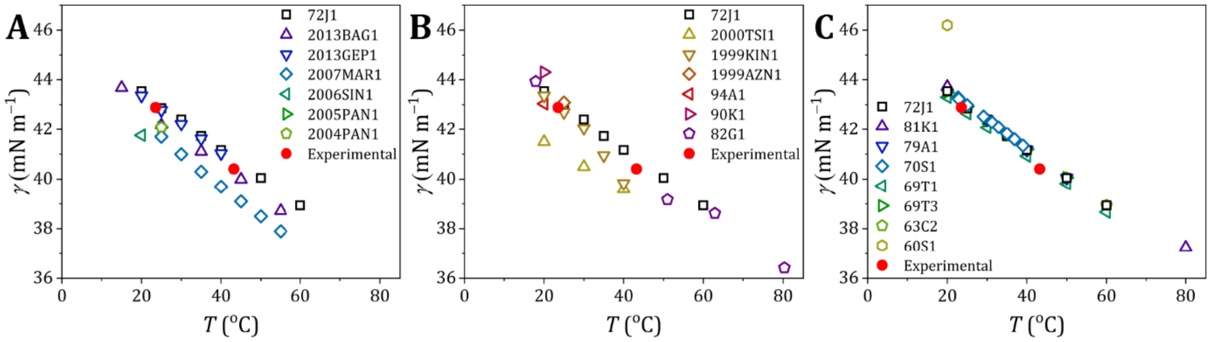

Figure S9: Surface tension of DMSO as measured experimentally (solid red circles) and reported by Jasper (72J1[56]) compared to literature values as taken from Landolt-Börnstein[5] and the associated supplements[6-7] (open symbols) for different temperatures. References from **A** 2004–2013: 2013BAG1[68], 2013GEP1[69], 2007MAR1[26], 2006SIN1[70], 2005PAN1[71], 2004PAN1[72]; **B** 1982–2000: 2000TSI1[73], 1999KIN1[74], 1999AZN1[75] 94A1[76], 90K1[77], 82G1[78]; **C** 1960–1981: 81K1[79], 79A1[80], 70S1[81], 69T1[82], 69T3[83], 63C1[84], 60S1[85].

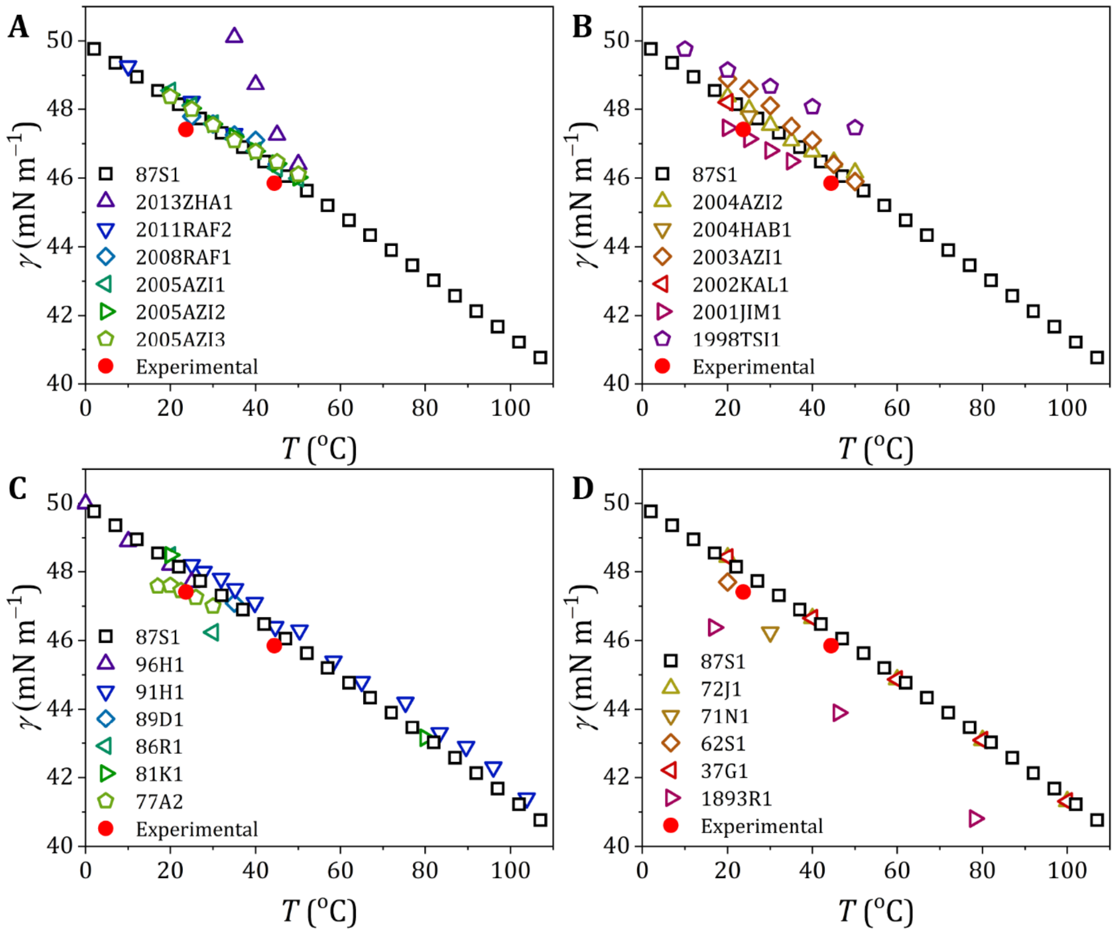

Figure S10: Surface tension of ethylene glycol as measured experimentally (solid red circles) and recommended by Dechema (87S1[98]) compared to literature values as taken from Landolt-Börnstein[5] and the associated supplements[6-7] (open symbols) for different temperatures. References from **A** 2005–2013: 2013ZHA1[86], 2011RAF1[11], 2008RAF1[87], 2005AZ1[88] (same data also presented in 2005AZI5[89]), 2005AZI2[90], 2005AZI3[91]; **B** 1998–2004: 2004AZI2[92], 2004HAB1[33], 2003AZI1[93], 2002KAL1[39], 2001JIM1[94], 1998TSI1[50]; **C** 1977–1996 96H1[95], 91H1[96], 89D1[97], 86R1[55], 81K1[79], 77A2?; 1893–1972: 72J1[56], 71N1[99], 62S1[100-101], 37G1[102], 1893R1[64]. Reference 77A2 is irrecoverable from the information provided.

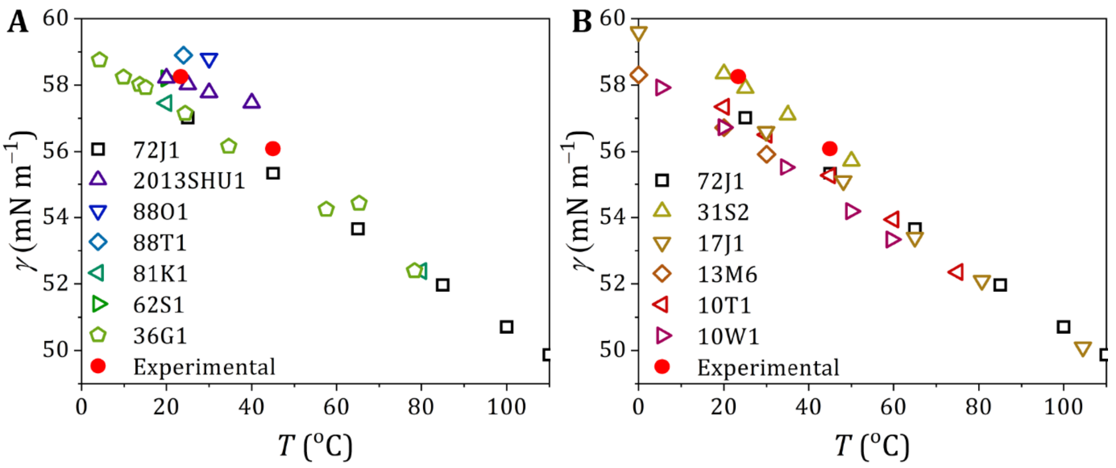

Figure S11: Surface tension of formamide as measured experimentally (solid red circles) and reported by Jasper (72J1[56]) compared to literature values as taken from Landolt-Börnstein[5] and the associated supplements[6-7] (open symbols) for different temperatures. References from **A** 1936–2013: 2013SHU1[103], 88O1[?], 88T1[104], 81K1, 72J1[56], 62S1[100-101], 36G1[105]; **B** 1910–1931: 31S2[106], 17J1[62], 13M6[107], 10T1[108]. Reference 88O1 does not deal with formamide.

SI-8: Surface tension data for the apolar liquids

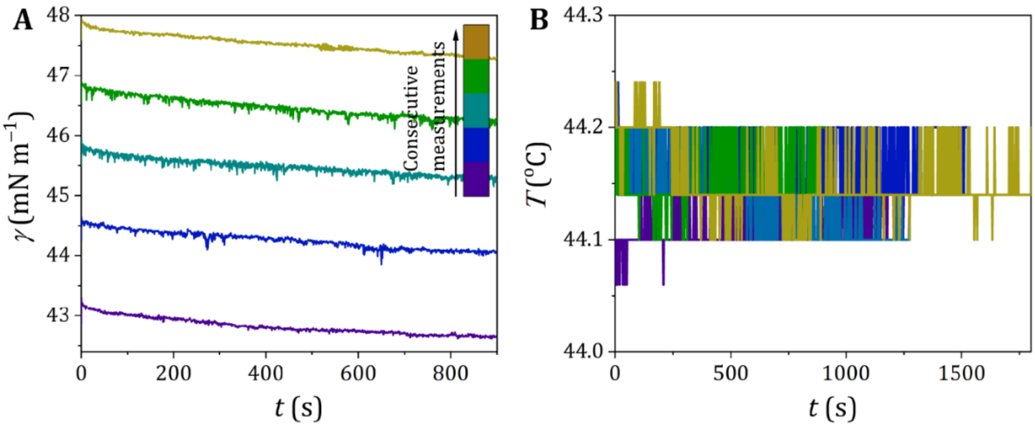

Figure S12: Surface tension measurements of diiodomethane at T = 44.2 °C. **A.** Surface tension measurements as function of time. **B.** Corresponding temperature profiles.

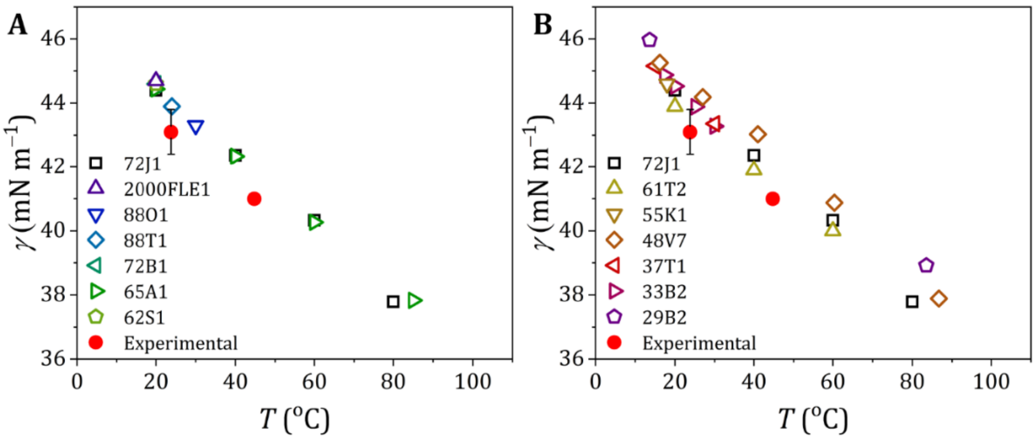

Figure S13: Surface tension of bromonaphthalene as measured experimentally (solid red circles) and reported by Jasper (72J1[56]) compared to literature values as taken from Landolt-Börnstein[5] and the associated supplements[6-7] (open symbols) for different temperatures. References from **A** 1962–2000: 2000FLE1[109], 88O1?, 88T1[104], 72B1?, 65A1?, 62S1[100-101]; **B** 1929–1961: 61T1[110], 55K1[111], 48V7?, 37T1[112], 33B2[113], 29B2[114]. References 65A1 and 48V7 are irrecoverable from the information provided. References 88O1 and 72B1 do not deal with bromonaphthalene.

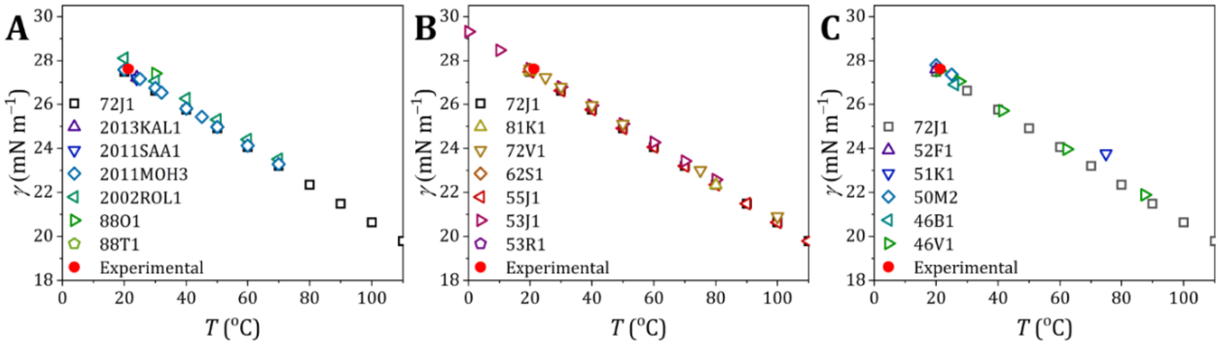

Figure S14: Surface tension of hexadecane as measured experimentally (solid red circles) and reported by Jasper (72J1[56]) compared to literature values as taken from Landolt-Börnstein[5] and the associated supplements[6-7] (open symbols) for different temperatures. References from **A** 1988–2013: 2013KAL1[115], 2011SAA1[116], 2011MOH3[117], 2002ROL1[118], 88O1?, 88T1[104]; **B** 1953–1981: 81K1, 62S1[100-101], 55J1[56], 53R1[119], 53J1[120]; **C** 1946–1952: 52F1[121], 51K1[122], 50M2?, 46B1[123], 46V1[124]. Reference 50M2 is irrecoverable from the information provided. Reference 88O1 does not deal with hexadecane.

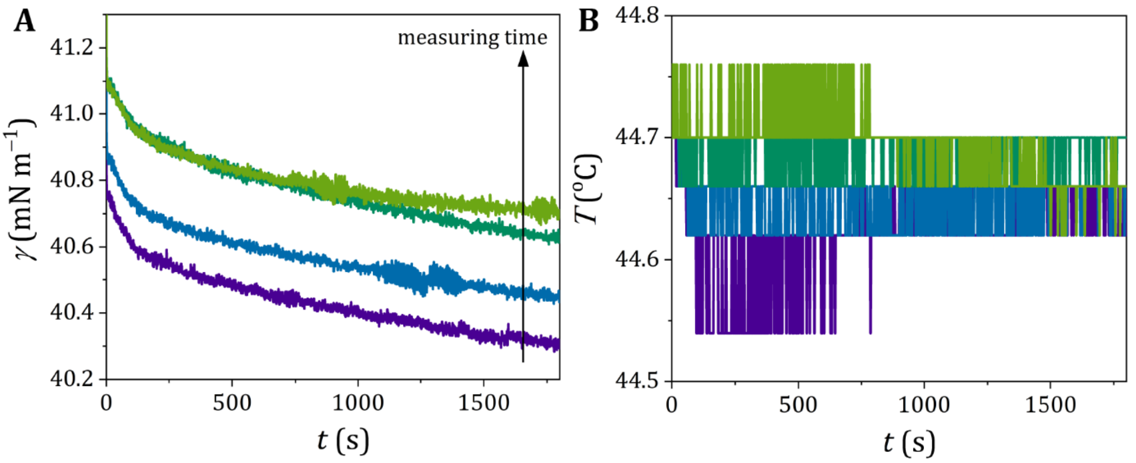

Figure S15: Surface tension measurements of 1-bromonaphthalene at *T* = 44.7 °C. A. Surface tension measurements as function of time. **B.** Corresponding temperature profiles.

SI-9: Chromatography paper experiments

As partial wetting of the platinum-iridium plate was obtained for diiodomethane, chromatography paper was used as an alternative, as suggested, amongst many others, by Partridge *et al*.[125]

To make the paper plates, a roll of chromatography paper (2 cm × 100 m, 1 CHR, Whatman) with a measured thickness of 0.18 mm was used. From this roll, rectangles with a length between 10 and 17 mm were cut as straight as possible using a paper cutter. These pieces of paper were then soaked in the liquid of interest for at least 10 min. Before data acquisition, the paper plate was hung above the liquid surface, with the cut edge facing the liquid using a clamp that was cleaned with ethanol and MilliQ water.

As an initial test for the reliability of this method, measurements with water were performed (Figure S16).

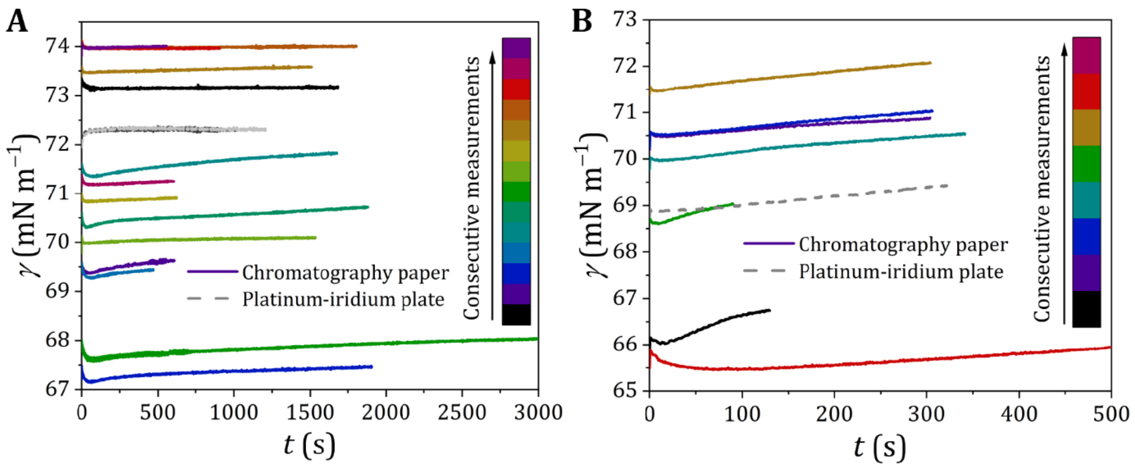

Figure S16: Use of chromatography paper to determine the surface tension. The surface tension was measured as a function of time at (**A**) 25 °C and (**B**) 45 °C. Each color represents a new measurement. The solid lines indicate the measurements that were performed with chromatography paper, the dashed lines indicate the measurements that were performed with the platinum-iridium plate. Note that the measurements with the platinum-iridium plate overlap.

For both the measurements that were performed at 25 °C as well as those performed at 45 °C, the measurements that were executed with chromatography paper display quite some deviation between the individual measurements. The measurements that were executed with the platinum-iridium plate, however, do show a great deal of overlap between separate measurements. Filter or chromatography paper is often advocated, in particular for non-wetting liquids but seems to be much less effective for wetting fluids. In any case, because of the swelling of the paper, the method will need a calibration with a liquid with a well-known surface tension.

SI-10: An explanation for the diiodomethane data discrepancy

In his overview Jasper[56] states that for diiodomethane the maximum bubble pressure method was used. Checking the original reference (Grzeskowiak *et al.*[126]) it appeared that, most likely, the capillary rise method was employed. These authors refer to an earlier paper in which it was stated that only for those liquids which did not appear to wet glass (and for those cases where an independent check was required, whatever that means) the maximum bubble pressure method was employed. Grzeskowiak *et al.*[126] do not mention that diiodomethane did not wet the glass capillary. Körösi *et al.*[79] (81K1) (capillary rise) report 50.88 mN m^{-1} at 20 °C, note the large difference with the Jasper data and state that diiodomethane decomposes above 40 °C. They also stated clearly that diiodomethane wets the capillary (Pyrex glass). A similar (but independently determined) value of 51.4 mN m^{-1} is reported by Fletcher and Nichols[109]. It appears that for thoroughly baked out fused silica the contact angle θ for diiodomethane is about 0°, but that exposure to (humid) air induces a non-zero contact angle converging to $\theta \sim 40°$ given sufficient time, typically ~3 h (Schrader[127]). Similarly, for (non-specified) glass slides and non-specified storage conditions $\theta \sim 49°$ was reported (Ozkan[128]) as well as advancing and receding angles of ~56° and 29° (Comte[129]) and ~48° and ~43° (Chibowski[130]). Grzeskowiak *et al.*[126] indicated that they did dry their glassware at 100 °C for 1 h, but that is certainly insufficient to obtain $\theta = 0°$. Therefore, it seems likely that θ in their case was not zero. Assuming $\theta = 41.2°$ would bring their value of $\gamma = 66.7$ mN m^{-1} to $\gamma = 50.88$ mN m^{-1} (the Körösi *et al.*[79] value), while $\theta = 39.6$ results in $\gamma = 51.4$ mN m^{-1} (the Fletcher and Nichols[109] value).

SI-11: Data for the surface tension calculation of solutions

In Table S11 the necessary data for the calculation of the surface tension of solutions of diiodomethane with iodine and 1-bromonaphthalene with bromine according to the considerations of Kaptay[131-132] are given. The interchange energy Ω is given by

$$\Omega = -\left\{\left[\frac{\Delta_{\text{vap}}H_1 - RT}{V_1}\right]^{\frac{1}{2}} - \left[\frac{\Delta_{\text{vap}}H_2 - RT}{V_2}\right]^{\frac{1}{2}}\right\}^2 \qquad \text{(S4)}$$

where $\Delta_{\text{vap}}H_j$ and V_j are the enthalpy of vaporization and molar volume of component j, respectively, and RT has its usual meaning. In Figure S17 and Table S11 the results for the diluted regime are plotted. The molar surface area ω is calculated as $\omega = N_A^{1/3}(V_m)^{2/3}$ with N_A Avogadro's number and V_m the molar volume. For the references referring to the surface

tensions, see the main manuscript. Thermodynamic data are varying somewhat in the literature, are mainly taken from the NIST Chemistry WebBook and, if necessary, extrapolated to room temperature.

Table S11: Data for the surface tension calculation according to Kaptay.

	M (g mol^{-1})	ρ (g cm^{-3})	V_m (cm^3 mol^{-1})	ω (m^2 mol^{-1})	γ (mN m^{-1})	$\Delta_{vap}H$ (kJ mol^{-1})	Ω (J m^{-3})
CH_2I_2	267.82	3.325	80.55	1.58×10^5	50.8	45.6	–1.96×10^7
I_2	253.81	4.933	51.45	5.15×10^5	46.9	41.6	
$C_{10}H_7Br$	207.07	1.48	139.3	1.40×10^5	44.4	56.0	–1.25×10^7
Br_2	159.81	3.103	51.50	5.15×10^5	41.8	30.0	

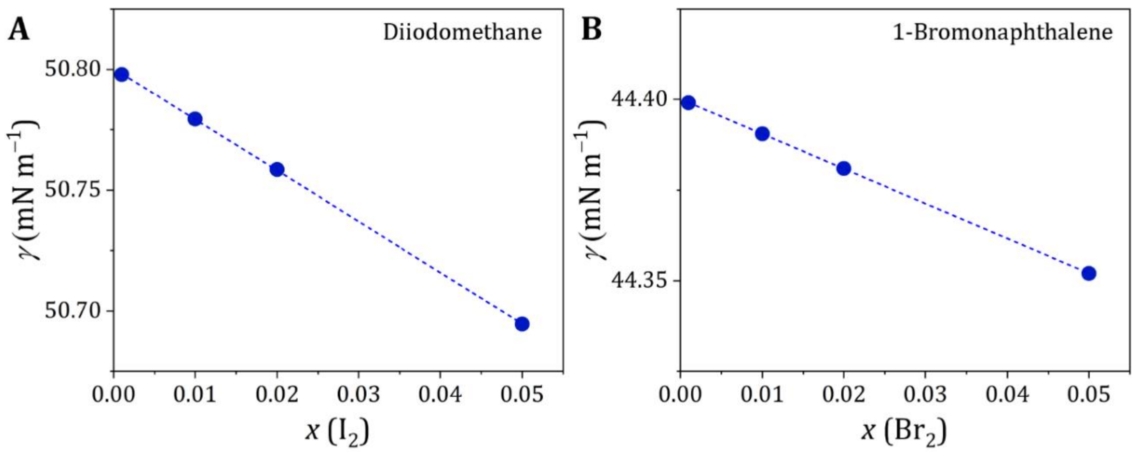

Figure S17: The change in surface tension of diluted solutions of (**A**) iodine in diiodomethane, described by $\Delta\gamma_{LV}$ (mN m^{-1}) = 50.8 – 2.1x, and (**B**) bromine in 1-bromonaphthalene, described by $\Delta\gamma_{LV}$ (mN m^{-1}) = 44.4 – 1.0x. The dots represent the calculated values and the dotted line represents the linear fit, both with R^2 =1.0.

References

[1] H. Kato, Karl Fischer reagents technical manual, *Mitsubishi Chemical Corporation, Japan*, **1999**,

[2] P. Bruttel, R. Schlink, Water determination by Karl Fischer titration, *Metrohm monograph*, **2003**, 8 (026), 50003.

[3] A. M. Mood, F. A. Graybill, D. C. Boes, *Introduction to the Theory of Statistics*, McGraw-Hill, Singapore **1974**.

[4] M. G. Kendall, A. Stuart, *The Advanced Theory of Statistics, Vol. 1, Distribution Theory*, Charles Griffin, London **1963**.

[5] C. Wohlfarth, B. Wohlfarth, *Surface Tension of Pure Liquids and Binary Liquid Mixtures*, Springer, **1997**.

[6] C. Wohlfarth, in *Landolt-Bornstein - Group IV Physical Chemistry 16: Surface Tension of Pure Liquids and Binary Liquid Mixtures*, Vol. 16 (Ed: M. D. Lechner), Springer Materials, Berlin Heidelberg **2008**.

[7] C. Wohlfarth, in *Landolt-Bornstein - Group IV Physical Chemistry 16: Surface Tension of Pure Liquids and Binary Liquid Mixtures*, Vol. 16 (Ed: M. D. Lechner), Springer Materials, Berlin Heidelberg **2016**.

[8] T. Petrova, R. B. Dooley, in *Proceedings of the International Association for the Properties of Water and Steam*, 1994.
[9] S. K. Begum, R. J. Clarke, M. S. Ahmed, S. Begum, M. A. Saleh, Densities, viscosities, and surface tensions of the system water+ diethylene glycol, *J. Chem. Eng. Data*, **2011**, 56 (2), 303-306.
[10] L. Blanco, O. Vargas, A. Suárez, Effect of temperature on the density and surface tension of aqueous solutions of HMT, *J. Therm. Anal. Calorim.*, **2011**, 104 (1), 101-104.
[11] A. A. Rafati, A. Bagheri, M. Najafi, Surface tension of non-ideal binary and ternary liquid mixtures at various temperatures and p=81.5kPa, *J. Chem. Thermodyn.*, **2011**, 43 (3), 248-254.
[12] A. V. Rayer, K. Z. Sumon, A. Henni, P. Tontiwachwuthikul, Physicochemical properties of {1-methyl piperazine (1)+ water (2)} system at T=(298.15 to 343.15) K and atmospheric pressure, *J. Chem. Thermodyn.*, **2011**, 43 (12), 1897-1905.
[13] F. Suárez, C. M. Romero, Apparent molar volume and surface tension of dilute aqueous solutions of carboxylic acids, *J. Chem. Eng. Data*, **2011**, 56 (5), 1778-1786.
[14] S. A. Kelayeh, A. H. Jalili, C. Ghotbi, M. Hosseini-Jenab, V. Taghikhani, Densities, viscosities, and surface tensions of aqueous mixtures of sulfolane+ triethanolamine and sulfolane+ diisopropanolamine, *J. Chem. Eng. Data*, **2011**, 56 (12), 4317-4324.
[15] H. K. Manchanda, M. Singla, A. Khosla, T. Singh, T. J. Trivedi, A. Kumar, Volumetric and surface properties of aqueous mixtures of polyethers at T=(298.15, 308.15, and 318.15) K, *J. Chem. Eng. Data*, **2011**, 56 (5), 2669-2676.
[16] M. Mohsen-Nia, H. Rasa, S. Naghibi, Experimental and theoretical study of surface tension of n-pentane, n-heptane, and some of their mixtures at different temperatures, *J. Chem. Thermodyn.*, **2010**, 42 (1), 110-113.
[17] M. Sadeghi, V. Taghikhani, C. Ghotbi, Measurement and correlation of surface tension for single aqueous electrolyte solutions, *Int. J. Thermophys.*, **2010**, 31 (4), 852-859.
[18] C. M. Romero, E. Jiménez, F. Suárez, Effect of temperature on the behavior of surface properties of alcohols in aqueous solution, *J. Chem. Thermodyn.*, **2009**, 41 (4), 513-516.
[19] E. Álvarez, D. Gómez-Díaz, M. D. La Rubia, J. Navaza, Surface Tension of Aqueous Binary Mixtures of 2-(Methylamino)ethanol and 2-(Ethylamino)ethanol and Aqueous Ternary Mixtures of These Amines with Triethanolamine or N-Methyldiethanolamine from (293.15 to 323.15) K., *J. Chem. Eng. Data*, **2008**, 53 1048.
[20] A. García-Abuín, D. Gómez-Díaz, J. M. Navaza, I. Vidal-Tato, Surface tension of aqueous solutions of short n-alkyl-2-pyrrolidinones, *J. Chem. Eng. Data*, **2008**, 53 (11), 2671-2674.
[21] C. M. Romero, M. S. Paéz, Surface tension of aqueous solutions of alcohol and polyols at 298.15 K, *Phys. Chem. Liq.*, **2006**, 44 (1), 61-65.
[22] S. Enders, H. Kahl, J. Winkelmann, Surface tension of the ternary system water+ acetone+ toluene, *J. Chem. Eng. Data*, **2007**, 52 (3), 1072-1079.
[23] C. Romero, M. S. Páez, J. A. Miranda, D. J. Hernández, L. E. Oviedo, Effect of temperature on the surface tension of diluted aqueous solutions of 1, 2-hexanediol, 1, 5-hexanediol, 1, 6-hexanediol and 2, 5-hexanediol, *Fluid Phase Equilib.*, **2007**, 258 (1), 67-72.
[24] I. M. Lampreia, Â. F. Santos, M. J. A. Barbas, F. J. Santos, M. L. Matos Lopes, Changes in aggregation patterns detected by diffusion, viscosity, and surface tension in water+ 2-(diethylamino) ethanol mixtures at different temperatures, *J. Chem. Eng. Data*, **2007**, 52 (6), 2388-2394.

[25] J. Li, M. Mundhwa, A. Henni, Volumetric properties, viscosities, refractive indices, and surface tensions for aqueous Genosorb 1753 solutions, *J. Chem. Eng. Data*, **2007**, 52 (3), 955-958.
[26] S. A. Markarian, A. M. Terzyan, Surface tension and refractive index of dialkylsulfoxide+ water mixtures at several temperatures, *J. Chem. Eng. Data*, **2007**, 52 (5), 1704-1709.
[27] R. Belda, J. Herraez, O. Diez, A study of the refractive index and surface tension synergy of the binary water/ethanol: influence of concentration, *Phys. Chem. Liq.*, **2005**, 43 (1), 91-101.
[28] D. Gómez-Díaz, J. M. Navaza, Surface behavior of aqueous solutions of pyrrolidine and piperidine, *J. Chem. Eng. Data*, **2004**, 49 (5), 1406-1409.
[29] Y. Maham, A. Chevillard, A. E. Mather, Surface thermodynamics of aqueous solutions of morpholine and methylmorpholine, *J. Chem. Eng. Data*, **2004**, 49 (3), 411-415.
[30] X.-X. Li, Y.-X. Liu, X.-H. Wei, Density, viscosity, and surface tension at 293.15 K and liquid− liquid equilibria from 301.15 K to 363.15 K under atmospheric pressure for the binary mixture of diethylene glycol diethyl ether+ water, *J. Chem. Eng. Data*, **2004**, 49 (4), 1043-1045.
[31] A.-P. Hyvärinen, H. Lihavainen, K. Hautio, T. Raatikainen, Y. Viisanen, A. Laaksonen, Surface tensions and densities of sulfuric acid+ dimethylamine+ water solutions, *J. Chem. Eng. Data*, **2004**, 49 (4), 917-922.
[32] P. Bauduin, L. Wattebled, S. Schroedle, D. Touraud, W. Kunz, Temperature dependence of industrial propylene glycol alkyl ether/water mixtures, *J. Mol. Liq.*, **2004**, 115 (1), 23-28.
[33] K. Habrdová, Š. Hovorka, L. Bartovská, Concentration Dependence of Surface Tension for Very Dilute Aqueous Solutions of Organic Nonelectrolytes, *J. Chem. Eng. Data*, **2004**, 49 (4), 1003-1007.
[34] M. L. Kijevcanin, I. Ribeiro, A. Ferreira, I. Fonseca, Water+ esters+ methanol: experimental data, correlation and prediction of surface and interfacial tensions at 303.15 K and atmospheric pressure, *Fluid Phase Equilib.*, **2004**, 218 (1), 141-148.
[35] E. Álvarez, Á. Cancela, R. Maceiras, J. M. Navaza, R. Táboas, Surface tension of aqueous binary mixtures of 1-amino-2-propanol and 3-amino-1-propanol, and aqueous ternary mixtures of these amines with diethanolamine, triethanolamine, and 2-amino-2-methyl-1-propanol from (298.15 to 323.15) K, *J. Chem. Eng. Data*, **2003**, 48 (1), 32-35.
[36] A. Kumar, V. Mohandas, P. Ghosh, Experimental Surface Tensions and Derived Surface Properties of Binary Mixtures of Water+ Alkoxyethanols (C1E m, m= 1, 2, 3) and Water+ Ethylene Glycol Dimethyl Ether (C1E1C1) at (298.15, 308.15, and 318.15) K, *J. Chem. Eng. Data*, **2003**, 48 (5), 1318-1322.
[37] B. Santos, A. Ferreira, I. Fonseca, Surface and interfacial tensions of the systems water+ n-butyl acetate+ methanol and water+ n-pentyl acetate+ methanol at 303.15 K, *Fluid Phase Equilib.*, **2003**, 208 (1-2), 1-21.
[38] U. Domanska, M. K. Kozlowska, M. Rogalski, Solubilities, partition coefficients, density, and surface tension for imidazoles plus octan-1-ol or plus water or plus n-decane, *J. Chem. Eng. Data*, **2002**, 47 (3), 456-466.
[39] G. Kalies, P. Bräuer, A. Schmidt, U. Messow, Calculation and Prediction of Adsorption Excesses on the Ternary Liquid Mixture/Air Interface from Surface Tension Measurements, *J. Colloid Interface Sci.*, **2002**, 247 (1), 1-11.
[40] T. Matsumoto, T. Nakano, H. Fujii, M. Kamai, K. Nogi, Precise measurement of liquid viscosity and surface tension with an improved oscillating drop method, *Phys. Rev. E.*, **2002**, 65 (3), 031201.

[41] J.-W. Lee, J.-S. Kim, H. Lee, Y.-W. Lee, Y.-S. Baek, H.-S. Pang, Surface tension and refractive index of (lithiumbromide+ water+ 1, 3-propanediol), *J. Chem. Thermodyn.*, **2001**, 33 (11), 1527-1534.
[42] Y. Maham, A. Mather, Surface thermodynamics of aqueous solutions of alkylethanolamines, *Fluid Phase Equilib.*, **2001**, 182 (1-2), 325-336.
[43] J. Gliński, G. Chavepeyer, J. K. Platten, Surface properties of diluted aqueous solutions of 3-picoline, *Colloids Surf. Physicochem. Eng. Aspects*, **2001**, 178 (1-3), 207-212.
[44] I. Tsuyumoto, H. Uchikawa, A high-performance and simplified quasi-elastic laser scattering method using homodyne detection in beam divergence, *Anal. Chem.*, **2001**, 73 (10), 2366-2368.
[45] Z. Yuan, K. Herold, Surface tension of pure water and aqueous lithium bromide with 2-ethyl-hexanol, *Appl. Therm. Eng.*, **2001**, 21 (8), 881-897.
[46] J.-W. Lee, S.-B. Park, H. Lee, Densities, surface tensions, and refractive indices of the water+ 1, 3-propanediol system, *J. Chem. Eng. Data*, **2000**, 45 (2), 166-168.
[47] J. Glinski, G. Chavepeyer, J.-K. Platten, Surface properties of diluted aqueous solutions of 1, 2-pentanediol, *J. Chem. Phys.*, **1999**, 111 (7), 3233-3236.
[48] S. Nath, Surface Tension of Nonideal Binary Liquid Mixtures as a Function of Composition, *J. Colloid Interface Sci.*, **1999**, 209 116-122.
[49] E. Alvarez, R. Rendo, B. Sanjurjo, M. Sanchez-Vilas, J. M. Navaza, Surface tension of binary mixtures of water+ N-methyldiethanolamine and ternary mixtures of this amine and water with monoethanolamine, diethanolamine, and 2-amino-2-methyl-1-propanol from 25 to 50 C, *J. Chem. Eng. Data*, **1998**, 43 (6), 1027-1029.
[50] N. G. Tsierkezos, I. E. Molinou, Thermodynamic properties of water+ ethylene glycol at 283.15, 293.15, 303.15, and 313.15 K, *J. Chem. Eng. Data*, **1998**, 43 (6), 989-993.
[51] C. M. Kinart, W. J. Kinart, A. Kolasiński, Acetonitryle-water binary mixtures and their assumed internal structures, *Phys. Chem. Liq.*, **1998**, 35 (4), 201-208.
[52] E. Álvarez, G. Vázquez, M. Sánchez-Vilas, B. Sanjurjo, J. M. Navaza, Surface tension of organic acids+ water binary mixtures from 20 C to 50 C, *J. Chem. Eng. Data*, **1997**, 42 (5), 957-960.
[53] G. Vázquez, E. Alvarez, J. M. Navaza, R. Rendo, E. Romero, Surface tension of binary mixtures of water+ monoethanolamine and water+ 2-amino-2-methyl-1-propanol and tertiary mixtures of these amines with water from 25 C to 50 C, *J. Chem. Eng. Data*, **1997**, 42 (1), 57-59.
[54] T. Mainzer-Althof, D. Woermann, Surface tension of a binary liquid mixture in the vicinity of its critical point, *Ber. Bunsenges. Phys. Chem.*, **1997**, 101 (7), 1014-1018.
[55] J. A. Riddick, W. B. Bunger, T. K. Sakano, *Organic Solvents: Physical Properties and Methods of Purification. Fourth Edition*, John Wiley and Sons, New York, NY, **1986**.
[56] J. J. Jasper, The Surface Tension of Pure Liquid Compounds, *J. Phys. Chem. Ref. Data*, **1972**, 1 (4), 841-1009.
[57] G. Gittens, Variation of surface tension of water with temperature, *J. Colloid Interface Sci.*, **1969**, 30 (3), 406-412.
[58] L. Volyak, *Temperature Dependence of the Surface Tension of Water*, US Atomic Energy Commission, Division of Technical Information, available from the Clearinghouse for Federal Scientific and Technical Information, National Bureau of Standards, US Department of Commerce, Springfield, Va., **1971**.
[59] L. Volyak, ISSLEDOVANIE TEMPERATURNOI ZAVISIMOSTI POVERKHNOSTNOGO NATYAZHENIYA VODY, *Dokl. Akad. Nauk SSSR*, **1950**, 74 (2), 307-310.
[60] W. Grant, W. Darch, S. Bowden, W. Jones, The surface tension and viscosity of solutions of uranyl salts, *J. Phys. Chem.*, **1948**, 52 (7), 1227-1236.

[61] P. Rehbinder, Surface activity and adsorptive power. II. Water as a surface-active material, *Z. Phys. Chem.*, **1926**, 121 103-126.
[62] F. M. Jaeger, Über die Temperaturabhängigkeit der molekularen freien Oberflächenenergie von Flüssigkeiten im Temperaturbereich von − 80 bis + 1650° C, *Z. anorg. allg. Chem.*, **1917**, 101 (1), 1-214.
[63] J. L. R. Morgan, A. M. McAfee, THE WEIGHT OF A FALLING DROP AND THE LAWS OF TATE. IX. THE DROP WEIGHTS OF THE ASSOCIATED LIQUIDS, WATER, ETHYL ALCOHOL, METHYL ALCOHOL AND ACETIC ACID; AND THE SURFACE TENSIONS AND CAPILLARY CONSTANTS CALCULATED FROM THEM, *J. Am. Chem. Soc.*, **1911**, 33 (8), 1275-1290.
[64] W. Ramsay, J. Schields, The Molecular Complexity of Liquids, *J. Chem. Soc., Trans.*, **1893**, 63 1089-1109.
[65] W. Ramsay, J. Shields, XIII. The variation of molecular surface-energy with temperature, *Philos. Trans. R. Soc. Lond. A.*, **1893**, (184), 647-673.
[66] S. Bi, G. Zhao, J. Wu, Surface Tension of Diethyl Ether, Diisopropyl Ether, and Dibutyl Ether, *J. Chem. Eng. Data*, **2010**, 55 (4), 1523-1526.
[67] A. Mulero, I. Cachadiña, A. Vegas, Recommended Correlations for the Surface Tension of 80 Esters, *J. Phys. Chem. Ref. Data*, **2021**, 50 (3), 033106.
[68] A. Bagheri, A. Abolhasani, A. Moghadasi, A. Nazari-Moghaddam, S. Alavi, Study of surface tension and surface properties of binary systems of DMSO with long chain alcohols at various temperatures, *J. Chem. Thermodyn.*, **2013**, 63 108-115.
[69] M. Geppert-Rybczyńska, J. K. Lehmann, J. Safarov, A. Heintz, Thermodynamic surface properties of [BMIm][NTf2] or [EMIm][NTf2] binary mixtures with tetrahydrofuran, acetonitrile or dimethylsulfoxide, *J. Chem. Thermodyn.*, **2013**, 62 104-110.
[70] M. Singh, Surface tension and viscosity measurements of liquids with the survismeter: a single instrumental unit, *Phys. Chem. Liq.*, **2006**, 44 (5), 579-584.
[71] A.-h. Pan, J.-h. Yao, Z.-g. Chen, X.-y. Wu, Determination of Lincomycin in Blood by High Performance Liquid Chromatography/Atmospheric Pressure Chemical lonization/Ion Trap Mass Spectrometry [J], *Acta Sci. Nat. Univ. Sunyatseni*, **2005**, 5 (3), 46.
[72] C. Pan, Q. Ke, G. Ouyang, X. Zhen, Y. Yang, Z. Huang, Excess Molar Volumes and Surface Tensions of Trimethylbenzene with Tetrahydrofuran Tetrachloromethane and Dimethyl Sulfoxide at 298.15 K, *J. Chem. Eng. Data*, **2004**, 49 (6), 1839-1842.
[73] N. G. Tsierkezos, A. E. Kelarakis, M. M. Palaiologou, Densities, Viscosities, Refractive Indices, and Surface Tensions of Dimethyl Sulfoxide + Butyl Acetate Mixtures at (293.15, 303.15, and 313.15) K, *J. Chem. Eng. Data*, **2000**, 45 (2), 395-398.
[74] C. M. Kinart, W. J. Kinart, A. Bald, The Measurements of the Surface Tension of Mixtures of Dimethyl Sulfoxide with Methyl, Ethyl and Propyl Alcohols, *Phys. Chem. Liq.*, **1999**, 37 (4), 317-321.
[75] S. B. Aznarez, M. Á. Postigo, A. Martins, Pressão interna e refração molar dos sistemas dimetilsulfóxido+ tiofeno, dimetilsulfóxido+ piridina a 298, 15K, *Eclet. Quim.*, **1999**, 24 17-27.
[76] S. Aznarez, L. Mussari, M. Postigo, Surface tension of the dimethylsulfoxide/thiophene system at 298.15 K, *Monatsh. Chem.*, **1994**, 125 (3), 241-246.
[77] I. Kreft, A. Paschke, J. Winkelmann, Surface tensions and interfacial tensions in three ternary systems, *Z. Phys. Chem.*, **1990**, 271 (1), 695-702.
[78] Y. V. Golubkov, R. I. Luchkina, L. L. Zolotova, V. T. Shibarshina, *Zh. Prikl. Khim.*, **1982**, 55
[79] G. Körösi, E. S. Kovats, Density and surface tension of 83 organic liquids, *J. Chem. Eng. Data*, **1981**, 26 (3), 323-332.

[80] D. K. Agarwal, R. Gopal, S. Agarwal, Surface tensions of binary liquid mixtures of some polar and nonpolar liquids with dimethyl sulfoxide (Me2SO), *J. Chem. Eng. Data*, **1979**, 24 (3), 181-183.
[81] W. E. Shipp, Surface tension of binary mixtures of several organic liquids at 25.deg, *J. Chem. Eng. Data*, **1970**, 15 (2), 308-311.
[82] E. Tommila, T. Autio, *Suom. Kemistil.*, **1969**, 42B 107.
[83] E. Tommila, Y. R, REFRACTIVE INDICES DENSITIES PARTIAL MOLAR VOLUMES SURFACE TENSIONS AND DIELECTRIC CONSTANTS OF DIMETHYL SULPHOX-IDE-ACETONE AND DIMETHYL SULPHOXIDE-DIOXAND MIXTURES, *Suom. Kemistil.*, **1969**, 42 (3), 90-&.
[84] H. L. Clever, C. C. Snead, Thermodynamics of Liquid Surfaces: The Surface Tension of Dimethyl Sulfoxide and some Dimethyl Sulfoxide-Acetone Mixtures, *J. Phys. Chem.*, **1963**, 67 918-920.
[85] H. L. Schläfer, W. Schaffernicht, Dimethylsulfoxyd als Lösungmittel für anorganische Verbindungen, *Angew. Chem.*, **1960**, 72 (17), 618-626.
[86] L. Zhang, C. Li, T. Huo, Z. Guo, Liquid Viscosities, Surface Tension and Viscous Flow Thermodynamics of Ethylene Glycol+ Water Mixtures at 308.15, 313.15, 318.15 and 323.15 K, *Asian J. Chem.*, **2013**, 25 (3),
[87] A. A. Rafati, E. Ghasemian, M. Abdolmaleki, Surface Properties of Binary Mixtures of Ethylene Glycol with a Series of Aliphatic Alcohols (1-Pentanol, 1-Hexanol, and 1-Heptanol), *J. Chem. Eng. Data*, **2008**, 53 (8), 1944-1949.
[88] S. Azizian, N. Bashavard, Surface thermodynamic functions of dilute solutions of methylcyclohexanols in ethylene glycol, *J. Colloid Interface Sci.*, **2005**, 286 (1), 349-354.
[89] S. Azizian, N. Bashavard, Surface Tensions of Dilute Solutions of Cycloheptanol in Ethylene Glycol, *J. Chem. Eng. Data*, **2005**, 50 (3), 1091-1094.
[90] S. Azizian, N. Bashavard, Equilibrium Surface Tensions of Benzyl Alcohol + Ethylene Glycol Mixtures, *J. Chem. Eng. Data*, **2005**, 50 (2), 709-712.
[91] S. Azizian, N. Bashavard, Surface properties of diluted solutions of cyclohexanol and cyclopentanol in ethylene glycol, *J. Colloid Interface Sci.*, **2005**, 282 (2), 428-433.
[92] S. Azizian, N. Bashavard, Surface thermodynamics of binary mixtures of ethylene glycol + cyclohexanol or cyclopentanol, *Colloids Surf. Physicochem. Eng. Aspects*, **2004**, 240 (1), 69-73.
[93] S. Azizian, M. Hemmati, Surface Tension of Binary Mixtures of Ethanol + Ethylene Glycol from 20 to 50 °C, *J. Chem. Eng. Data*, **2003**, 48 (3), 662-663.
[94] E. Jiménez, M. Cabanas, L. Segade, S. García-Garabal, H. Casas, Excess volume, changes of refractive index and surface tension of binary 1,2-ethanediol + 1-propanol or 1-butanol mixtures at several temperatures, *Fluid Phase Equilib.*, **2001**, 180 (1), 151-164.
[95] A. Horibe, S. Fukusako, M. Yamada, Surface tension of low-temperature aqueous solutions, *Int. J. Thermophys.*, **1996**, 17 (2), 483-493.
[96] B. C. Hoke, J. C. Chen, Binary aqueous-organic surface tension temperature dependence, *J. Chem. Eng. Data*, **1991**, 36 (3), 322-326.
[97] T. Daubert, R. Danner, **1989**.
[98] K. Stephen, H. Hildwein, DECHEMA: Frankfurt am Main, Germany, 1987.
[99] K. Nakanishi, T. Matsumoto, M. Hayatsu, Surface tension of aqueous solutions of some glycols, *J. Chem. Eng. Data*, **1971**, 16 (1), 44-45.
[100] E. Shafrin, W. Zisman, Effect of progressive fluorination of a fatty acid on the wettability of its adsorbed monolayer, *J. Phys. Chem.*, **1962**, 66 (4), 740-748.

[101] F. Schulman, W. A. Zisman, The spreading of liquids on low-energy surfaces. V. Perfluorodecanoic acid monolayers, *J. Colloid Sci.*, **1952**, 7 (5), 465-481.
[102] A. Gallaugher, H. Hibbert, Studies on Reactions Relating to Carbohydrates and Polysaccharides. LIV. The Surface Tension Constants of the Polyethylene Glycols and their Derivatives1, *J. Am. Chem. Soc.*, **1937**, 59 (12), 2514-2521.
[103] R. Shukla, A. Kumar, U. Srivastava, N. Awasthi, J. Pandey, Critical evaluation of surface tension of binary liquid mixtures from associated and nonassociated processes at various temperatures: an experimental and theoretical study, *Can. J. Phys.*, **2013**, 91 (3), 211-220.
[104] N. Tillman, A. Ulman, J. S. Schildkraut, T. L. Penner, Incorporation of phenoxy groups in self-assembled monolayers of trichlorosilane derivatives. Effects on film thickness, wettability, and molecular orientation, *J. Am. Chem. Soc.*, **1988**, 110 (18), 6136-6144.
[105] F. H. Getman, Cryoscopic studies of solutions in formamide. I, *Recl. Trav. Chim. Pays-Bas*, **1936**, 55 (3), 231-243.
[106] G. F. Smith, The Purification and Some Physical Constants of Formamide, *J. Chem. Soc.*, **1931**, 3257-3263.
[107] J. L. R. Morgan, E. C. Stone, THE WEIGHT OF A FALLING DROP AND THE LAWS OF TATE. XII. THE DROP WEIGHTS OF CERTAIN ORGANIC LIQUIDS AND THE SURFACE TENSIONS AND CAPILLARY CONSTANTS CALCULATED FROM THEM, *J. Am. Chem. Soc.*, **1913**, 35 (10), 1505-1524.
[108] W. E. S. Turner, E. W. Merry, *J. Chem. Soc.*, **1910**, 97
[109] P. D. I. Fletcher, R. J. Nicholls, Alkylbenzenes in Diiodomethane. A Novel, “Primitive” Micelle-Forming Surfactant System, *Langmuir*, **2000**, 16 (3), 1050-1056.
[110] A. P. Toropov, V. N. Chamaev, *Izv. Vyssg. Uchebn. Zav., Khim. Khim. Tekhnol.*, **1961**, 4
[111] F. Kozlenko, S. Miskidzhyan, FIZIKO-KHIMICHESKII ANALIZ SISTEMY ALLILOVOE GORCHICHNOE MASLO-ETILOVYI SPIRT, *Zh. Obshch. Khim.*, **1955**, 25 (1), 35-40.
[112] J. Timmermans, M. Hennaut-Roland, *J. Chim. Phys. Phys. Chim. Biol.*, **1937**, 34 693.
[113] F. Bartell, A. D. Wooley, Solid—Liquid—Air Contact Angles and their Dependence upon the Surface Condition of the Solid, *J. Am. Chem. Soc.*, **1933**, 55 (9), 3518-3527.
[114] S. S. Bhatnagar, B. Singh, *J. Indian Chem. Soc.*, **1929**, 6 264.
[115] A. Kalantarian, S. M. I. Saad, A. W. Neumann, Accuracy of surface tension measurement from drop shapes: The role of image analysis, *Adv. Colloid Interface Sci.*, **2013**, 199-200 15-22.
[116] S. M. I. Saad, Z. Policova, A. W. Neumann, Design and accuracy of pendant drop methods for surface tension measurement, *Colloids Surf. Physicochem. Eng. Aspects*, **2011**, 384 (1), 442-452.
[117] M. Mohsen-Nia, Measurement and modelling of surface tensions of systems containing n-hexadecane, n-heptane and n-pentane, *Phys. Chem. Liq.*, **2011**, 49 (5), 608-614.
[118] L. I. Rolo, A. I. Caço, A. J. Queimada, I. M. Marrucho, J. A. P. Coutinho, Surface Tension of Heptane, Decane, Hexadecane, Eicosane, and Some of Their Binary Mixtures, *J. Chem. Eng. Data*, **2002**, 47 (6), 1442-1445.
[119] F. D. Rossini, *Selected Values of Physical and Thermodynamic Properties of Hydrocarbons and Related Compounds: Comprising the Tables of the American Petroleum Institute Research Project 44 Extant as of December 31, 1952*, American Petroleum Institute, **1953**.
[120] J. J. Jasper, E. R. Kerr, F. Gregorich, The Orthobaric Surface Tensions and Thermodynamic Properties of the Liquid Surfaces of the n—Alkanes, C5 to C28, *J. Am. Chem. Soc.*, **1953**, 75 (21), 5252-5254.

[121] H. W. Fox, C. H. Chrisman, The Ring Method of Measuring Surface Tension for Liquids of High Density and Low Surface Tension, *J. Phys. Chem.*, **1952**, 56 (2), 284-287.
[122] H. T. Kempf, J. Reitstötter, Zur Oberflächenspannung der Paraffine, *Kolloid-Z.*, **1951**, 123 (1), 38-38.
[123] W. C. Bigelow, D. L. Pickett, W. A. Zisman, Oleophobic monolayers: I. Films adsorbed from solution in non-polar liquids, *J. Colloid Sci.*, **1946**, 1 (6), 513-538.
[124] A. I. Vogel, 38. Physical properties and chemical constitution. Part IX. Aliphatic hydrocarbons, *J. Chem. Soc.*, **1946**, 133-139.
[125] M. Partridge, F. Davis, S. W. James, S. P. Higson, R. P. Tatam, A solution to the slow stabilisation of surface pressure sensors based on the Wilhelmy method, *Matters*, **2017**,
[126] R. Grzeskowiak, G. H. Jeffery, A. I. Vogel, 921. Physical properties and chemical constitution. Part XXXI. Polymethylene dichlorides, dibromides, di-iodides, and dicyanides, *J. Chem. Soc.*, **1960**, 4728-4731.
[127] M. E. Schrader, Ultra-high vacuum techniques in the measurement of contact angles: Methylene iodide on glass, *J. Colloid Interface Sci.*, **1968**, 27 (4), 743-750.
[128] O. Ozkan, H. Y. Erbil, Interpreting contact angle results under air, water and oil for the same surfaces, *Surf. Topogr. Metrol. Prop.*, **2017**, 5 (2), 024002.
[129] F. Comte, D. Sage, presented at Makromolekulare Chemie. Macromolecular Symposia **1989**.
[130] E. Chibowski, M. Jurak, Comparison of contact angle hysteresis of different probe liquids on the same solid surface, *Colloid. Polym. Sci.*, **2013**, 291 (2), 391-399.
[131] G. Kaptay, Partial Surface Tension of Components of a Solution, *Langmuir*, **2015**, 31 (21), 5796-5804.
[132] J. Korozs, G. Kaptay, Derivation of the Butler equation from the requirement of the minimum Gibbs energy of a solution phase, taking into account its surface area, *Colloids Surf. Physicochem. Eng. Aspects*, **2017**, 533 296-301.
